# Reversion of resistance to oxaliplatin by inhibition of p38 MAPK in colorectal cancer cell lines: involvement of the calpain / Nox1 pathway

**DOI:** 10.18632/oncotarget.21780

**Published:** 2017-10-10

**Authors:** Mathieu Chocry, Ludovic Leloup, Hervé Kovacic

**Affiliations:** ^1^ Aix-Marseille Université, INSERM, CRO2 UMR_S 911, Marseille 13385, France

**Keywords:** NADPH oxidase, calpain, colorectal cancer, oxaliplatin, chemoresistance

## Abstract

Oxaliplatin is a major treatment for metastatic colorectal cancer, however its effectiveness is greatly diminished by the development of resistances. Our previous work has shown that oxaliplatin efficacy depends on the reactive oxygen species (ROS) produced by Nox1. In this report, we investigated Nox1 involvement in the survival mechanisms of oxaliplatin resistant cell lines that we have selected. Our results show that basal ROS production by Nox1 is increased in resistant cells. Whereas the transitory Nox1-dependent production of superoxide contributes to the cytotoxicity of oxaliplatin in sensitive cells, oxaliplatin treatment of resistant cells leads to a decrease in the production of superoxide associated with an increase of H_2_O_2_ and a decreased cytotoxicity of oxaliplatin. We have shown that calpains regulate differently Nox1 according to the sensitivity of the cells to oxaliplatin. In sensitive cells, calpains inhibit Nox1 by cleaving NoxA1 leading to a transient ROS production necessary for oxaliplatin cytotoxic effects. In contrast, in resistant cells calpain activation is associated with an increase of Nox1 activity through Src kinases, inducing a strong and maintained ROS production responsible for cell survival. Using a kinomic study we have shown that this overactivation of Nox1 results in an increase of p38 MAPK activity allowing the resistant cells to escape apoptosis. Our results show that the modulation of Nox1 activity in the context of anticancer treatment remains complex. However, a strategy to maximize Nox1 activation while inhibiting the p38 MAPK-dependent escape routes appears to be an option of choice to optimize oxaliplatin efficiency.

## INTRODUCTION

Colorectal cancer (CRC) became a major cancer due to its increasing frequency and its mortality. It is currently the third most common cancer in the world, with nearly 1.4 million new cases diagnosed in 2012 [1]. The average 5-year survival rate is about 52%, however major disparities are observed, depending on the CRC stages [1]. A quarter of the patients are diagnosed at the metastatic stage and 50% will develop metastasis [2]. At this stage, the survival rate is only 13.1% in the USA (National Cancer Institute) and less than 10% in Europe (6.6% in England, National Cancer Intelligence Network, 2009, and 8.1% in France, Institut National du Cancer, 2012).

At metastatic stage, CRC is usually treated with multidrug chemotherapies, such as FOLFIRI and FOLFOX regimens. These first-line treatments, constituted of 5-fluorouracil (5-FU), folinic acid and irinotecan or oxaliplatin (L-OHP), have equivalent efficacies which remain unfortunately low due to the therapeutic escape of tumor cells. Oxaliplatin is a third-generation platinum-based alkylating agent which induces the formation of intra- and inter-strand bridges between guanines leading to the inhibition of DNA replication and synthesis. The oxaliplatin monotherapy has a very low activity with an objective response rate not exceeding 10%, while the combination of oxaliplatin with 5-FU and folinic acid strongly improves the treatment efficiency, increasing the objective response rate up to 58% [3]. However, around 50% of the patients develop resistances to this chemotherapy protocol leading to treatment failure [4]. It is thus critical to identify the actors of these resistances, particularly to oxaliplatin, to improve the treatment efficiency and to predict the tumor cell response.

Our previous works have highlighted the crucial role played by Nox1 and the reactive oxygen species (ROS) in the response of CRC cells to oxaliplatin [5]. In tumor cells, the main sources of ROS are the mitochondrial respiratory chain and NADPH oxidases (Nox) [6]. The five identified Nox isoforms (from Nox1 to Nox5) are non-mitochondrial multi-protein enzymes associated with the plasma membrane. They produce superoxide ions by oxidizing the NADPH coenzyme with the help of associated proteins. Nox1 isoform is associated with four proteins that are necessary for its activity: p22phox, Rac1, NoxO1 (NADPH oxidase organizer 1) and NoxA1 (NADPH oxidase activator 1). Nox1 is localized in the invagination of the plasma membrane (caveolae) as well as on the endosome surface, allowing the enzyme to produce extracellular and endoplasmic ROS. Nox1 has numerous functions and regulates many fundamental physiological processes as well as pathological phenomena. NADPH oxidases present tissue-specific expression patterns and Nox1 isoform is strongly expressed in the colon and generally overexpressed in colorectal tumor cells [7]. Due to its implication in CRC cell response to oxaliplatin, it is crucial to have a better understanding of Nox1 activity and role, however the regulation of this enzyme remains unclear.

In this work, we have studied the implication of calpains in the regulation of Nox1 activity. The calpain family consists of 15 calcium-dependent cysteine proteases (from calpain 1 to calpain 16), which are classified according to their tissue expression. We can distinguish ubiquitous calpains such as calpain 1 and calpain 2, and tissue-specific calpains such as calpain 9 which is expressed mainly in the digestive tract (and under-expressed in CRC cell). Calpains 1 and 2 activities are mainly regulated by calcium and by their specific inhibitor, calpastatin [8]. Calpain 2 is also regulated by ERK and PKA phosphorylations and by its localization at the membrane [9–11]. These calpains are broad-spectrum enzymes, cleaving cytoskeletal proteins (such as talin, vinculin, etc.), transcription factors (p53, c-fos…) or enzymes (caspases, Rho A, Rac…) [8, 12]. The diversity of these substrates explains the large number of physiological and pathological phenomena in which calpains are involved. This is notably the case for cancers since several studies have shown an involvement of calpains in tumor invasion and in angiogenesis but also in the response to chemotherapy. However, their roles seem to be antinomic according to cancers. Indeed, calpains are involved in the cytotoxic effects of genistein and trastuzumab in breast cancer, and of cisplatin in melanoma and ovarian cancer [13–15], while calpain 2 is implicated in the resistance of CRC cells to irinotecan [16].

Based on these data and on our previous work, we have studied the implication of Nox1 and calpains in the resistance of CRC cells to oxaliplatin. After establishing oxaliplatin-resistant colorectal cancer lines, we observed that these cell lines showed an increase in Nox1 and calpain activity. In sensitive cells, calpains inhibit Nox1 activity by cleaving NoxA1 leading to the transient production of ROS necessary for oxaliplatin cytotoxic effects. In contrast, in resistant cells the activation of calpains is responsible for the increase of Nox1 without inducing mortality under treatment with oxaliplatin. Kinomic approach allowed us to demonstrate the activation of an escape route to cell death secondary to the overactivation of Nox1 in resistant cells. Our results confirm that the production of ROS by Nox1 is necessary for the efficacy of oxaliplatin. However, in resistant cells, adaptive mechanisms are developed downstream of Nox1 to limit the oxaliplatin-induced cytotoxicity. Taken together, our data show that the new identified calpain/Nox1/p38 MAPK pathway could be an interesting therapeutic target to improve oxaliplatin-based treatment efficiency and a predictive marker of oxaliplatin resistance.

## RESULTS

### Selection of cells resistant to oxaliplatin

We selected oxaliplatin-resistant colorectal cancer cells by growing HT29-D4 and RKO cells in the presence of increasing concentrations of oxaliplatin (see Material and Methods section). The selection allowed us to obtain two different sub-lines for HT29-D4 that will be called HT29-D4 Rox1 (Rox1) and HT29-D4 Rox2 (Rox2) and two sub-lines for RKO (RKO Rox1 and RKO Rox2). These cells are considered resistant as they are able to grow in culture medium containing 2 μM of oxaliplatin, this concentration corresponding to the clinically relevant plasma concentration of patients treated with oxaliplatin. This 2 μM concentration was thus used in all our experiments.

**Figure 1:**
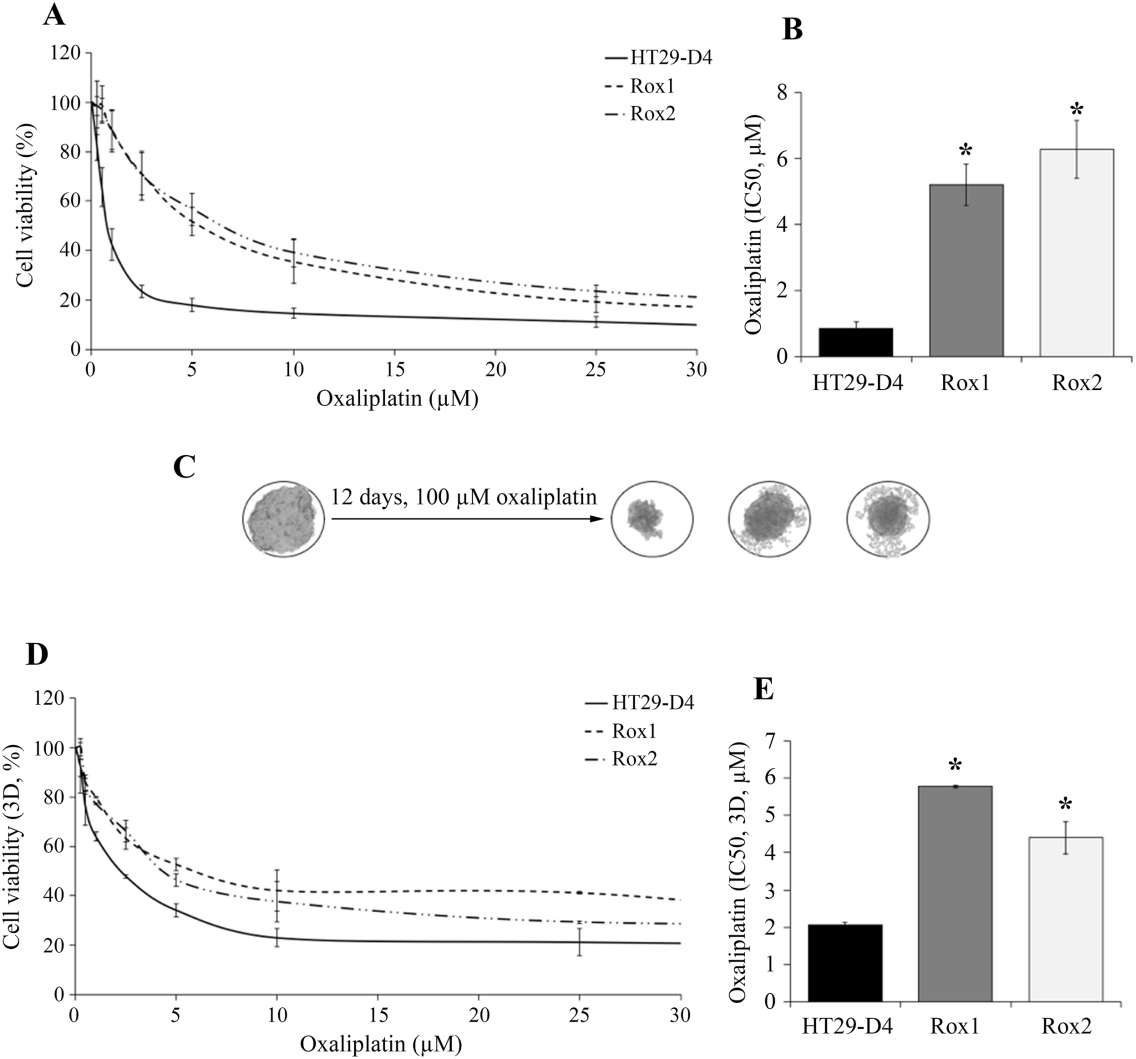
Validation of the resistance status of the selected cells The HT29-D4, HT29-D4-Rox1 (Rox1) and HT29-D4-Rox2 (Rox2) cells were submitted to a 72-hour cytotoxicity assay in 2 dimensions (2D, **A**) and in 3 dimensions (3D, spheroids, **D**). The IC50 of oxaliplatin were then calculated using the Chou and Talalay’s method in 2D (**B**) and 3D models (**E**). The effects of oxaliplatin on spheroids are illustrated in (**C)**. Asteriks indicate a statistical significance with *p*<0.05.

Cytotoxicity assays show that Rox1 and Rox2 cells are resistant to oxaliplatin in comparison to the sensitive HT29-D4 cells (Figure 1A-1B). Indeed, low oxaliplatin concentrations (0.25 and 0.5 μM, inferior to 2 μM) has no significant effects on the resistant cells, their survival rates remaining between 96 and 99%. On the opposite, the viability of sensitive cells is impacted, decreasing to 83% at 0.25 μM and 65% at 0.5 μM (Figure 1A). The IC_50_ of oxaliplatin is 0.8 ± 0.2 μM for HT29-D4 cells compared to 5.2 ± 0.6 μM for Rox1 and 6.3 ± 0.9 μM for Rox2 cells (*p*<0.05, Figure 1B). These significant differences confirm the resistance of our selected cells to oxaliplatin. Similar results were obtained with RKO cells (Supplementary Figure 1).

To get closer to the tumor environment, the cytotoxic effects of oxaliplatin were also studied in a 3D cell culture model. For HT29-D4 cells, the IC_50_ of oxaliplatin is 2.1 ± 0.1 μM, this value is increased to 5.8 ± 0.1 μM and 4.4 ± 0.4 μM for Rox1 and Rox2 cells, respectively (Figure 1D-1E). Spheroids growth in sensitive and resistant cell lines over time were presented in Supplementary Figure 2. These results confirmed the resistance of our cells, with similar profils in 2D and 3D experiments.

### Nox1 isoform is necessary for oxaliplatin cytotoxicity

**Figure 2:**
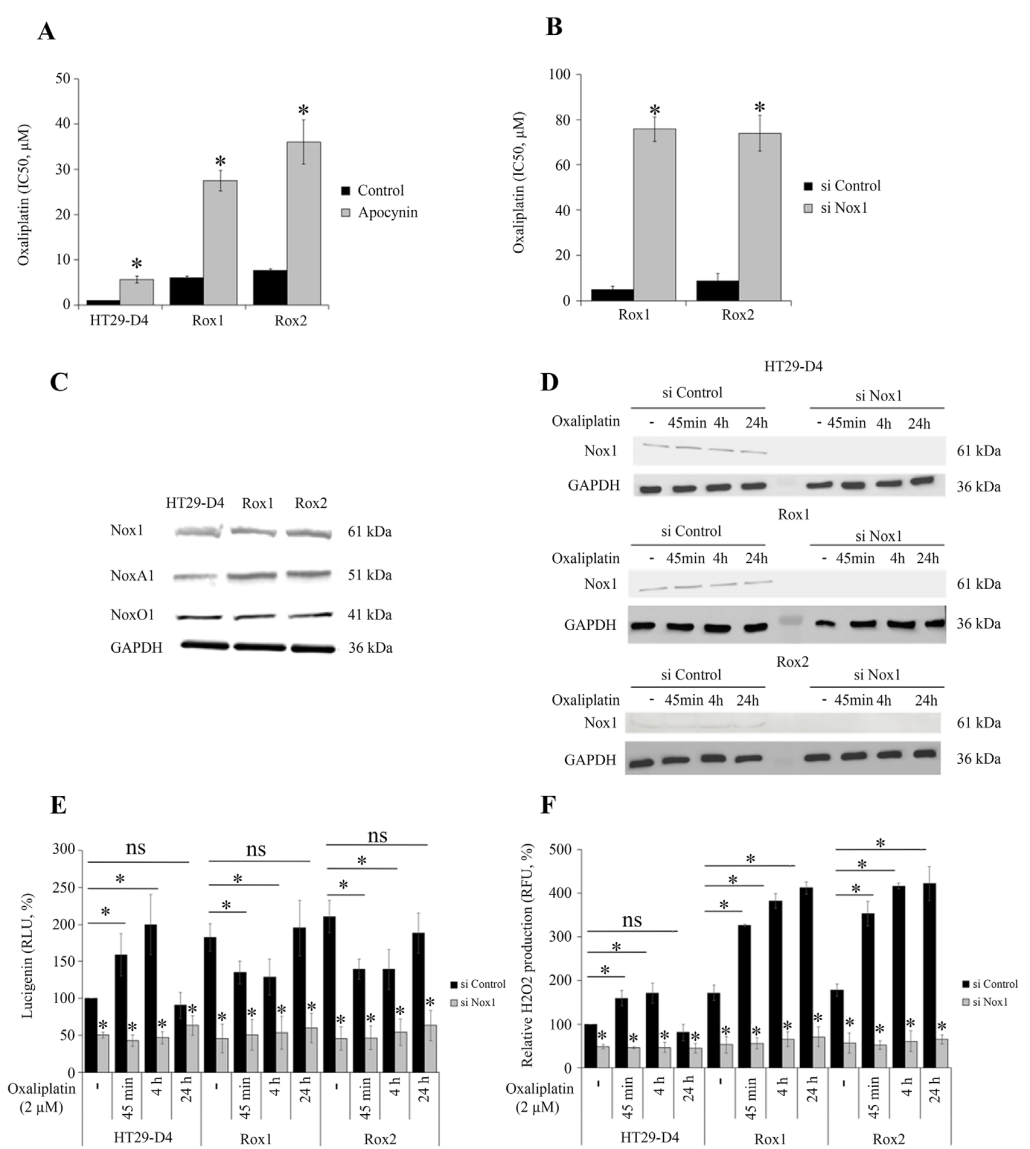
Implication of Nox1 in oxaliplatin-induced ROS production and cytotoxicity The effects of oxaliplatin on cell viability were studied with HT29-D4, Rox1 and Rox2 cells treated with apocynin **(A)** or transfected with control siRNA (si Control) or Nox1 specific siRNA (si Nox1) **(B)**. HT29-D4, Rox1 and Rox2 cells were lysed, and equal amounts of cellular proteins were processed for immunoblotting using the antibodies against Nox1, NoxA1, NoxO1 and GAPDH **(C)**. HT29-D4, Rox1 and Rox2 cells were transfected with control siRNA (si Control) and Nox1 specific siRNA (si Nox1). The cells were lysed, and equal amounts of cellular protein were processed for immunoblotting using the antibodies against Nox1 **(D)**. Transfected cells were also seeded in white 96-well plates to perform lucigenin assays (**E**) and in black 96-well plates to perform Amplex red assay **(F)**. These cells were treated with 2 μM oxaliplatin over time (- untreated, 45 minutes (45 min), 4 hours (4h) and 24 hours (24h)). Asteriks indicate a statistical significance with *p*<0.05.

Our previous works have shown that ROS produced by Nox1 are necessary for oxaliplatin-induced cytotoxicity [5]. Treatment of cells with apocynin (0.5 mM) confirmed that ROS production impacts oxaliplatin cytotoxicity in sensitive cells but also in resistant cells. Apocynin increased the IC_50_ of oxaliplatin for HT29-D4 from 0.9 ± 0.1 μM to 5.6 ± 0.7 μM (Figure 2A). The IC_50_of oxaliplatin for Rox1 cells was increased from 5.2 ± 0.6 μM to 27.5 ± 2.3 μM in the presence of this inhibitor (Figure 2A). Similar results were obtained with Rox2 cells (Figure 2A). The repression of Nox1 expression leads to a significant decrease of oxaliplatin efficiency, the IC_50_ increasing from 5.9 ± 0.5 μM for Rox1 cells transfected with control siRNA to 75.8 ± 5.0 μM with siRNA directed against Nox1. The IC_50_ for Rox2 cells were similarly modified increasing from 9.1 ± 1.4 μM to 74.0 ± 8.2 μM (Figure 2B). We have then compared the expression levels of the different proteins constituting Nox1 complex in these cells. Our results show no significant difference in the expression of Nox1 and NoxO1 between sensitive and resistant cells (Figure 2C). However, the expression of NoxA1 was increased by 31.5% and 32.9% in Rox1 and Rox2 cells, respectively (Figure 2C, Supplementary Figure 3C). The effects of oxaliplatin on Nox1 expression were also studied. As shown in Figure 2D, oxaliplatin has no significant effect on Nox1 expression in our resistant and sensitive cells.

We have then studied Nox1 activity by measuring the superoxide production. Our different cell lines were treated with oxaliplatin and the superoxide production was monitored over the time using lucigenin chemiluminescence. Our results show that in the absence of oxaliplatin superoxide production is strongly and significantly increased in our resistant cells, reaching 182.4 ± 18.7% for Rox1 cells and 210.0 ± 21.9% for Rox2 cells (*p*<0.05, Figure 2E). Similar data were obtained with RKO cells, the superoxide production increasing to 275.4 ± 24.8% for RKO Rox1 cells and to 275.0 ± 37.1% for RKO Rox2 cells (Supplementary Figure 4A). The repression of Nox1 expression using siRNA induced strong reductions of the superoxide production, showing clearly that Nox1 is the major source of superoxide production in our cells and that Nox1 is responsible for the increases observed in the resistant cells (Figure 2E).

We have then studied the effects of oxaliplatin on the superoxide production in our sensitive and resistant cells. The kinetic study of superoxide production shows that oxaliplatin stimulates significantly Nox1 activity in sensitive cells after 45 minutes and 4 hours of treatment, the superoxide production reaching 159.2 ± 28.7% and 199.8 ± 40.7%, respectively. Superoxide production is then reduced, returning to regular levels after 24 hours (Figure 2E). In resistant cells, opposite effects are observed. In Rox1 cells, superoxide production is significantly reduced by oxaliplatin, decreasing from 182.4 ± 18.7% to 135.0 ± 15.1% after 45 minutes and to 129.1 ± 24.3% after 4 hours. Similarly, the superoxide production of Rox2 cells decreased from 210.7 ± 21.9% to 139.7 ± 13.4% and 139.3 ± 26.6% when oxaliplatin is added for 45 minutes or 4 hours, respectively (Figure 2E). Like for sensitive cells, superoxide production returns to regular levels after 24 hours.

Extracellular H_2_O_2_ production was measured using Amplex Red fluorescence, in the same conditions than for superoxides (the H_2_O_2_ production of untreated HT29-D4 cells was set to 100%). In the absence of oxaliplatin, extracellular H_2_O_2_ production was strongly and significantly increased in our resistant cells, reaching 171.2 ± 17.6% for Rox1 cells and 178.0 ± 14.0% for Rox2 cells (*p*< 0.05, Figure 2F). The results obtained for the cells transfected with the siRNA against Nox1 show that Nox1 activity is required for the production of H_2_O_2_. Indeed, the inhibition of Nox1 expression reduces the H_2_O_2_ production by 50% in the sensitive cells and by more than 65% in the resistant ones. A kinetic study of oxaliplatin effects was also performed. The results show that the peroxide production is stimulated by oxaliplatin in sensitive cells for short time treatments, like it was observed for superoxide production previously. The production is then reduced and returns to regular levels after 24 hours. In resistant cells, oxaliplatin strongly stimulates the production of H_2_O_2_ after 45 minutes and 4 hours, as shown in Figure 2F. Unlike what was observed with sensitive cells, the peroxide production is maintained at high levels even after 24 hours of treatment.

Taken together, these data indicate that oxaliplatin stimulates Nox1 activity in sensitive cells to increase transiently ROS production and thus induce cell death. Our data also show that ROS production is overactivated and dysregulated in our resistant cells.

### Calpain activity and roles are modified in resistant cells in comparison to sensitive cells

**Figure 3:**
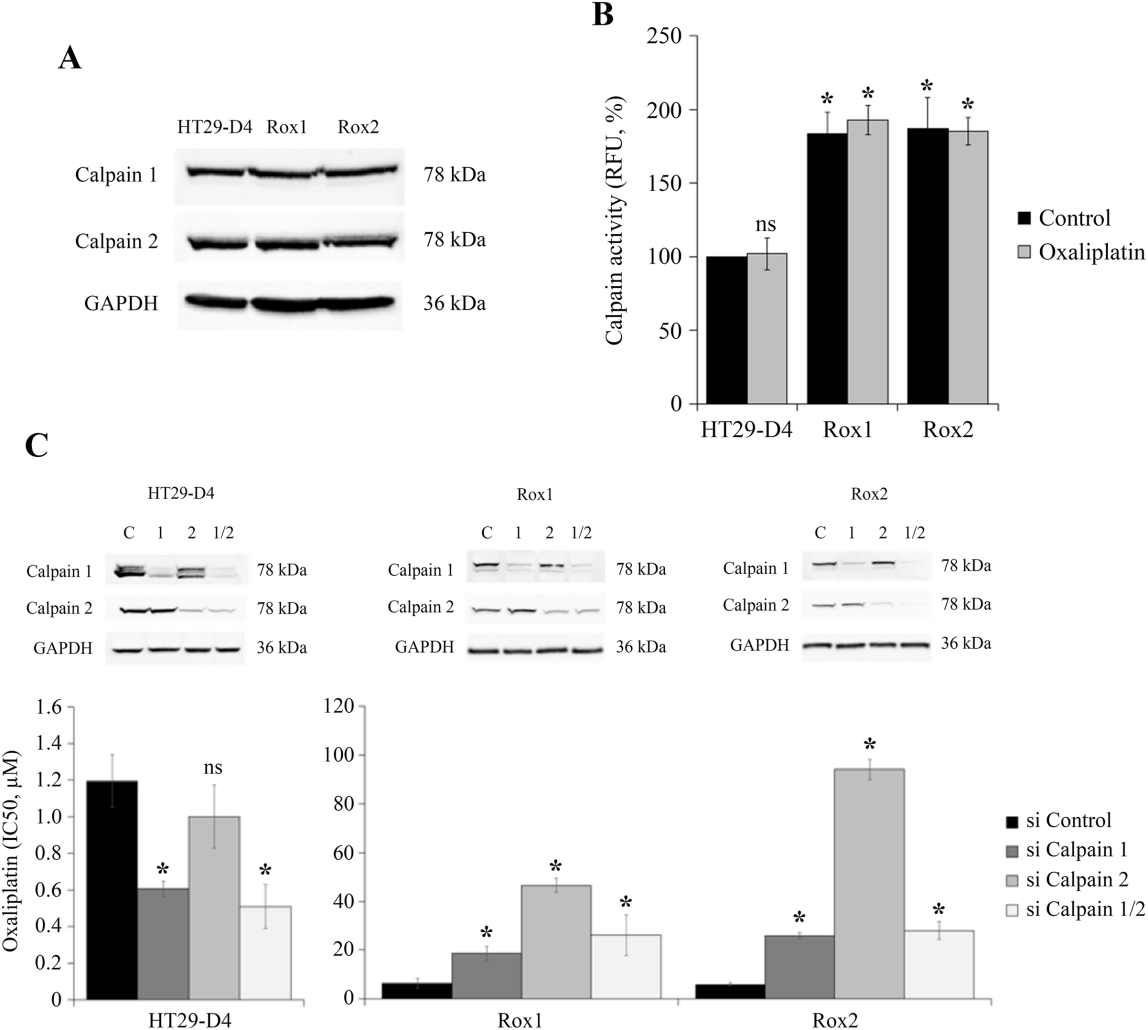
Study of calpain expression, activity and implication in oxaliplatin-induced cytotoxicity HT29-D4, Rox1 and Rox2 cells were lysed and equal amounts of proteins were processed for immunoblotting using the antibodies against calpain 1, calpain 2 and GAPDH **(A)**. HT29-D4, Rox1 and Rox2 cells were seeded in black 96-well plates to perform calpain activity assays with (Oxaliplatin) or without oxaliplatin (Control) **(B)**. HT29-D4, Rox1 and Rox2 cells were transfected with control siRNA (si Control), calpain-1 specific siRNA (si Calpain 1), calpain 2 specific siRNA (si Calpain 2) or both siRNAs (si Calpain 1/2). The cells were lysed and equal amounts of proteins were processed for immunoblotting using antibodies against calpain 1 and calpain 2 (C). The transfected cells were also seeded to perform 72-hour cytotoxicity assays **(C)**. Asteriks indicate a statistical significance with *p*<0.05.

Recent studies have identified calpain 2 as an actor of the resistance of colorectal cancer cells to irinotecan [16], we have thus studied the potential implication of ubiquitous calpains in the resistance to oxaliplatin. Firstly, the expression of calpains 1 and 2 was compared between sensitive and resistant cells. Our results show no significant difference in the expression level of these two proteases (Figure 3A, Supplementary Figure 5A-5B). However, as calpains are enzymes regulated notably by calcium and phosphorylations, their activity may not be correlated with their expression. Calpain activity was thus measured in our sensitive and resistant cells using the fluorescent substrate t-boc-LM-CMAC (calpain activity in HT29-D4 cells was fixed at 100%). Our results show a strong and significant stimulation of calpain activity in the resistant cells. Indeed, calpain activity reached 193.0 ± 9.8% for Rox1 cells and 185.1 ± 9.4% for Rox2 cells (Figure 3B). Oxaliplatin has no effect on calpain activity, in sensitive and in resistant cells. Very similar results were obtained with sensitive and resistant RKO cells (Supplementary Figure 4B). These data indicate that the stimulation of calpain activity observed in the resistant cells is constitutive and does not depend on the presence of oxaliplatin.

To identify the calpain isoform responsible for this increase of activity, the experiment was repeated using cells transfected with siRNAs directed against calpain 1 and/or 2. The efficacy of these siRNAs was confirmed and the underexpression was maintained after 96 hours (Figure 3C). The activity assays carried out with these transfected cells show that the two isoforms are equally involved in the increase of activity observed with the resistant cells and that there is no isoform specifically responsible for this phenomenon (Supplementary Figure 5C). To identify the potential roles played by calpains in the resistance to oxaliplatin, cytotoxicity assays were performed with HT29-D4, Rox1 and Rox2 cells under-expressing calpain 1 and/or 2. Our results obtained with the sensitive cells show that the repression of the expression of calpain 1 or both calpains reduces the IC_50_ of oxaliplatin in comparison to cells transfected with control siRNA (Figure 3D). Indeed, the IC_50_ of oxaliplatin in HT29-D4 cells was reduced from 1.2 ± 0.1 μM with control siRNA to 0.6 ± 0.1 μM with siRNA against calpain 1 and to 0.5 ± 0.1 μM with the siRNA directed against the two isoforms (Figure 3C). No significant difference was observed when repressing calpain 2 expression. On the opposite, in resistant cells our results show that the repression of calpain expression leads to an increase of the IC_50_ of oxaliplatin (Figure 3C). The IC_50_ values are increased from 6.4 ± 1.9 μM for Rox1 and 5.9 ± 0.6 μM for Rox2 cells, to 18.7 ± 2.9 μM and 26.0 ± 1.2 μM when calpain 1 expression is repressed. The inhibition of calpain 2 expression has stronger effects, the IC_50_ of oxaliplatin reaching 46.6 ± 2.9 μM for the Rox1 and 96.0 ± 4.1 μM for Rox2 (Figure 3C). The inhibition of the two calpains gives results close to those obtained with the siRNA directed against calpain 1.

Taken together these results show that calpains reduce oxaliplatin effects in the sensitive cells while they are required for oxaliplatin-induced cytotoxicity in the resistant cells.

### Calpain regulation involves calcium and PKC delta

We have then studied the activation of calpains to identify the actors responsible for the stimulation of calpain activity observed in the resistant cells. As shown in Figure 3A, the expression of the calpain isoforms is not responsible for the increased activity. It is well known that calpains can be activated by calcium and that calpain 2 activity is stimulated by ERK/MAPK pathway. We have thus studied these two activation pathways.

**Figure 4:**
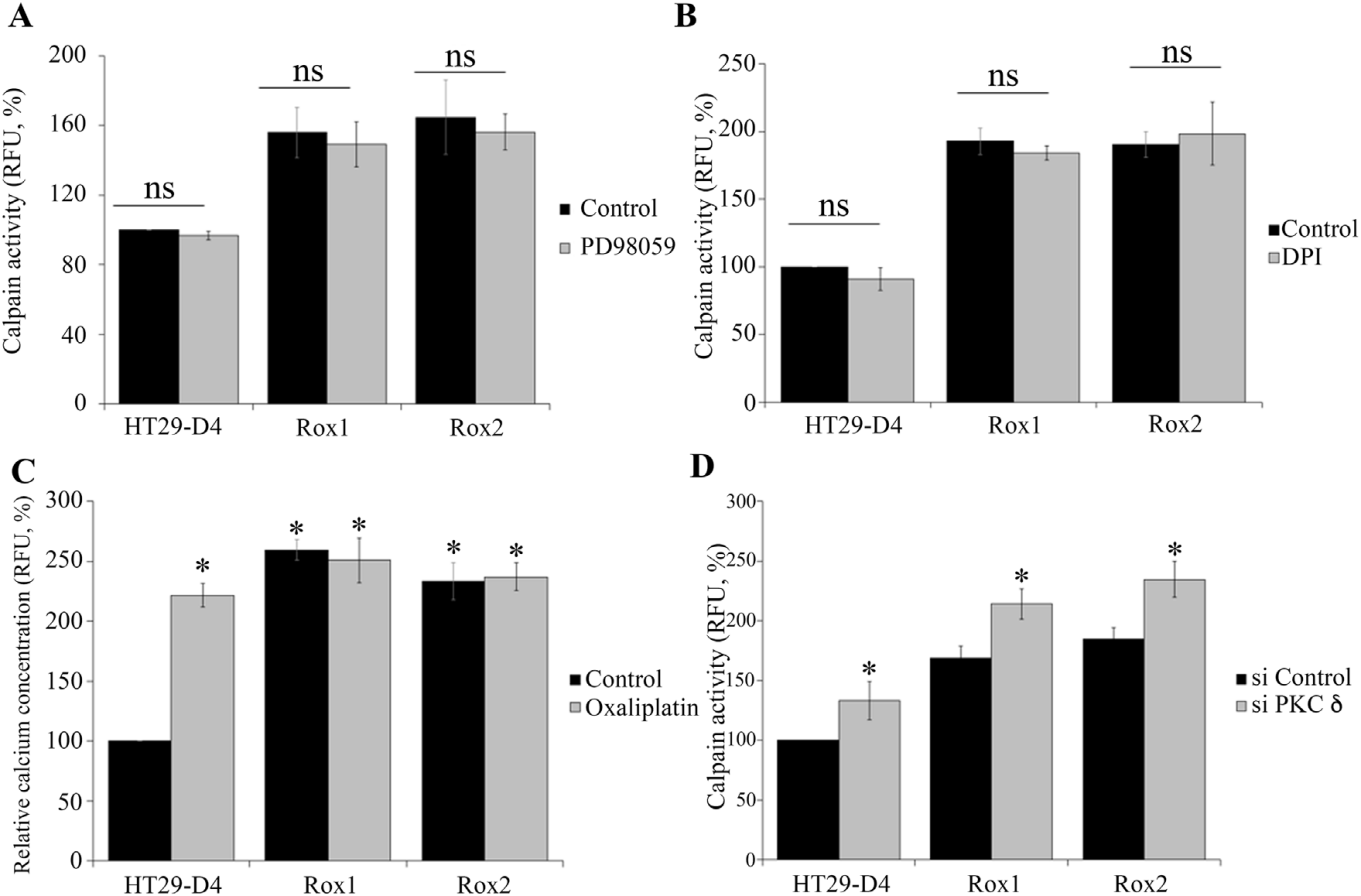
Regulation of calpain activity HT29-D4, Rox1 and Rox2 cells were seeded in black 96-well plates to perform calpain activity assays in the absence (control) or in the presence of a MEK inhibitor (PD98058, 2.5 μM) **(A)**. Calpain activity was measured like previously (A) with cells incubated in the absence (Control) or in the presence of an inhibitor of ROS (DPI, 5 μM) **(B)**. HT29-D4, Rox1 and Rox2 cells were treated with (Oxaliplatin, 2 μM) or without oxaliplatin (Control) and incubated with 10 μM of FURA-2-AM to measure the intracellular concentration of calcium (**C**). HT29-D4, Rox1 and Rox2 cells were transfected with control siRNA (si CTRL) or PKC d specific siRNA (si PKC delta) and seeded in black 96-well plates to perform calpain activity assays **(D)**. Asteriks indicate a statistical significance with *p*<0.05.

We have firstly performed calpain activity assays in our cells treated with or without PD98059, a specific ERK/MAPK pathway inhibitor. The results show that PD98059 has no significant effect on calpain activity in sensitive and resistant cells. Indeed, in sensitive cells PD98059 reduced calpain activity only from 100% to 96.8 ± 2.5%. In the same manner, the inhibitor reduced the activity from 155.8 ± 11.3% to 149.2 ± 13.1% in Rox1 cells and from 164.7 ± 12.9% to 156.1 ± 10.4% in Rox2 cells (Figure 4A). These data clearly indicate that the ERK/MAPK pathway is not responsible for the calpain activity increase observed in our oxaliplatin resistant cells.

We therefore carried out calpain activity assays with our HT29-D4, Rox1 and Rox2 cells, treated or not with DPI (10 μM), a specific inhibitor of Nox enzymes. As shown in Figure 4B, no significant effect on calpain activity was observed. The activity was only reduced from 100% to 90.9 ± 8.5% in sensitive cells, from 193.0 ± 9.8% to 184.2 ± 5.1% in Rox1 cells, and only slightly increased from 190.5 ± 9.4% to 198.5 ± 23.3% in Rox2 cells (Figure 4B). These data clearly indicate that ROS produced by Nox1 do not regulate calpain activity in our cells.

Intracellular calcium levels were then measured using FURA2-AM. Firstly, our results show clearly that oxaliplatin induces a strong increase of the intracellular calcium concentration in the sensitive HT29-D4 cells. The relative concentration was indeed increased from 100% to 221.7 ± 9.6% by oxaliplatin (Figure 4C). Our data show also that intracellular calcium levels are increased in the resistant cells and that oxaliplatin has no effect on calcium concentration in these cells. In Rox1 cells, the relative calcium concentration was measured at 221.7 ± 9.6% in the absence of oxaliplatin and 250.8 ± 18.4% in the presence of this chemotherapy agent (Figure 4C). Similarly, in Rox2 cells calcium concentration was not impacted by oxaliplatin, increasing only from 233.5 ± 15.4% to 236.9 ± 23.3%. These data show that the intracellular calcium concentration is constitutively increased in the cells resistant to oxaliplatin, and suggest that this increase could be responsible for calpain over-activation.

As shown in Figure 4D, the inhibition of PKCδ leads to an increase of calpain activity in both the sensitive and resistant cells. In HT29-D4, the activity (fixed at 100% for the control siRNA) was significantly increased to 133.1 ± 15.9% when PKCδ expression is repressed. Calpain activity was also increased from 168.9 ± 10.0% and 185.1 ± 9.3% to 214.1 ± 12.6% and 234.7 ± 14.9% in Rox1 and Rox2 cells, respectively (Figure 4D). These results demonstrate that PKCδ regulates negatively calpain activity.

### Calpains regulate Nox1 activity in both sensitive and resistant cells

We then investigated the existence of a potential regulation between calpains and Nox1.

**Figure 5:**
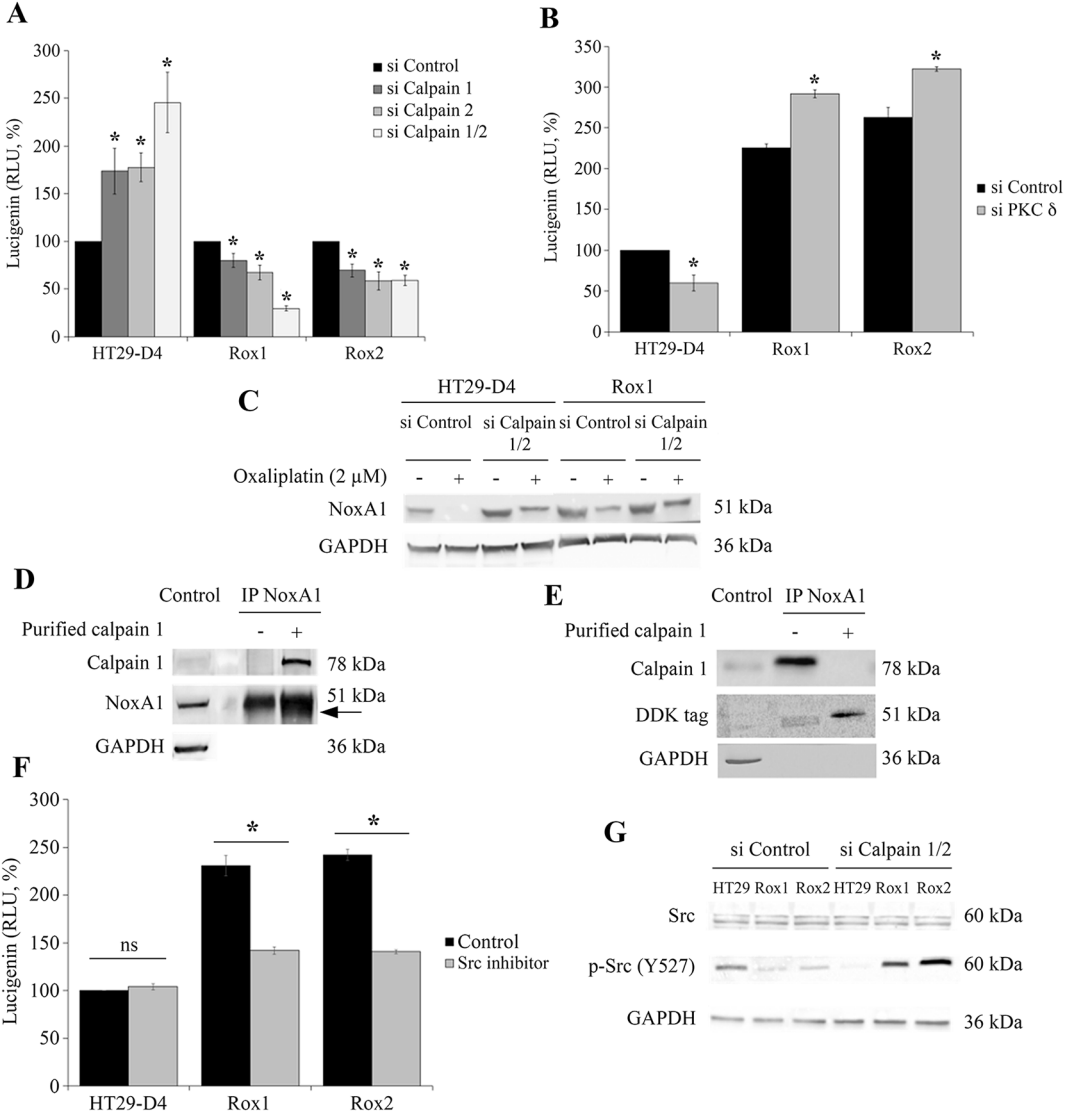
Regulation of Nox1 by calpain HT29-D4, Rox1 and Rox2 cells were transfected with control siRNA (si Control), calpain 1 specific siRNA (si Calpain 1), calpain 2 specific siRNA (si Calpain 2) or with both siRNA (si Calpain 1/2). The cells were seeded in white 96-well plates to perform lucigenin assays **(A)**, and in 6-well plates to perform Western blots after oxaliplatin treatment (2 μM) **(C)**. HT29-D4, Rox1 and Rox2 cells were transfected with control siRNA (si CTRL) or PKC d specific siRNA (si PKC delta) and seeded in white 96-well plates to perform lucigenin assays **(B)**. NoxA1 was immunoprecipitated from HT29-D4 lysates and incubated with purified calpain 1. The samples were then processed for immunoblotting using antibodies against calpain 1, NoxA1 and GAPDH (Control: total lysate before IP, **(D)**. HT29-D4 cells were transfected with a plasmid encoding NoxA1-DDK. The cells were lysed, and NoxA1 was immunoprecipitated (IP NoxA1) and incubated with purified calpain 1. The samples were then processed for immunoblotting using antibodies against calpain 1, DDK tag and GAPDH **(E)**. HT29-D4, Rox1 and Rox2 cells were seeded in white 96-well plates to perform lucigenin assays in the absence (Control) or in the presence of Src inhibitor (**F**). HT29-D4 (HT29), Rox1 and Rox2 cells were transfected with control siRNA (si Control), or both calpain 1 and calpain 2 specific siRNAs (si Calpain 1/2). The cells were then lysed and the proteins were processed for immunoblotting using antibodies against Src, p-Src (tyrosine 527) and GAPDH **(G)**. Asteriks indicate a statistical significance with *p*<0.05.

We have thus studied the effects of the inhibition of calpain expression on the production of ROS by Nox1. For this purpose, ROS measurements were performed using our sensitive and resistant cells transfected with siRNA directed against calpain 1 and/or 2. The ROS production of the cells transfected with the control siRNA was set at 100%. Interestingly, we have observed opposite regulations depending on the resistance status of our cells. In the sensitive cells, we have clearly observed that the inhibition of ubiquitous calpain expression leads to a strong and significant increase of the production of ROS. Indeed, the inhibition of calpain 1 and calpain 2 expression increases the production of ROS from 100% to 173.5 ± 24.1% and 177.6% ± 15.3%, respectively (Figure 5A). The repression of both calpain expression leads to a major peak of ROS production (245.5 ± 31.8%, Figure 5A). We have also confirmed that these increases of ROS production are depending on Nox1 activity (data not shown). Surprisingly, opposite effects were observed in resistant cells, as the down-regulation of calpain expression induced a strong inhibition of ROS production. In Rox1 cells, the repression of calpain 1 and calpain 2 reduced ROS production from 100% to 79.9 ± 7.6% and 67.2% ± 7.6%, respectively (Figure 5A). In Rox2 cells, we observed a 31% decrease of ROS production with the siRNA directed against calpain 1 and a 42% decrease with the siRNA directed against calpain 2. In the presence of both siRNA, the production was decreased to 29.6 ± 2.6% for Rox1 and 58.9 ± 5.6% for Rox2 (Figure 5A). As shown in Supplementary Figure 4C, the same regulations were observed in RKO cells.

As we have shown that PKCδ regulates negatively calpain activity, we have studied the effects of the inhibition of PKCδ expression on ROS production. Like with the siRNA directed against calpains, we have observed opposite effects in the sensitive and the resistant cells. A significant decrease in ROS production was observed in the sensitive cells, from 100% to 59.8 ± 9.7%, while in resistant cells the production was increased from 225.3± 5.2% to 291.9 ± 5.0% and from 262.7 ± 12.2% to 322.3 ± 2.7% for Rox1 and Rox2 cells, respectively (Figure 5B).

Taken together, these data prove the existence of a regulation of Nox1 activity by the two ubiquitous calpains, in both sensitive and resistant cells. However, this regulation is inverted according to the sensitivity of the cells to oxaliplatin, calpains repressing Nox1 activity in sensitive cells and stimulating this activity in resistant cells.

### Calpains inhibit Nox1 in sensitive cells through NoxA1 degradation

To characterize the regulation of Nox1 activity by calpains, we have studied the expression of the proteins constituting Nox1 complex, particularly the activator NoxA1. We have transfected sensitive and resistant cells with the siRNA directed against calpain 1 and calpain 2 and we have observed the effects of a 24-hour treatment with oxaliplatin on NoxA1 expression. The results show that oxaliplatin induces a strong decrease of NoxA1 expression in both the sensitive and the resistant cells. However, the effects of oxaliplatin are limited in the resistant cells in comparison to the sensitive cells, in which NoxA1 protein is not detectable after the oxaliplatin treatment (Figure 5C). The repression of ubiquitous calpain expression induces an increase of NoxA1 expression in the sensitive cells and limits the effects of oxaliplatin. In the resistant cells, the same phenomenon is observed at a weaker extent. These results suggest that calpains could regulate Nox1 through the degradation of NoxA1. To confirm this regulation, NoxA1 was purified by immunoprecipitation and then incubated in the presence of purified calpain 1. Our results show that the presence of calpain 1 induces the appearance of a second band around 49 kDa, very close to the band corresponding to the full length NoxA1 (51 kDa; Figure 5D). Calpains would thus cleave NoxA1 at its extremities, as predicted using the SVM prediction model (Supplementary Figure 6A). To characterize this cleavage, we have performed the same experiment using a NoxA1 protein modified by the addition of a DDK tag at its N-terminal extremity. The effects of the incubation of the immunoprecipitated NoxA1 with the purified calpain 1 were observed using an antibody directed against the DDK tag. The results are similar to those obtained using the endogenous NoxA1, showing two bands, the upper one corresponding to the full length NoxA1 at 51 kDa and the smaller one to the cleaved NoxA1 (Figure 5E). As the cleaved NoxA1-DDK is also observed around 49 kDa, we can thus conclude that calpains are cleaving NoxA1 at its C-terminal extremity. This C-terminal part is known to be involved in the binding of NoxO1, required for the activation of Nox1 (Supplementary Figure 6B).

### Calpains activate Nox1 in resistant cells through Src activation

The direct cleavage of NoxA1 by calpains is responsible for the repression of Nox1 activity in sensitive cells, however this cleavage is very limited in the resistant cells and cannot be responsible for the activation of Nox1 by calpains observed in these cells. Src is known to be a potential activator of Nox1, we have thus characterized the activation of Nox1 by calpains by studying Src implication.

We have firstly studied the potential involvement of Src kinases in the regulation of ROS production by using a specific inhibitor of these enzymes. Our results show that Src inhibitor has no significant effect on the production of ROS in the sensitive cells (production increased from 100% to 104.1 ± 3.2%), whereas significant inhibitions were observed in our resistant cells (Figure 5F). The production of ROS was decreased from 231.0 ± 10.5 to 141.8 ± 4.0% for Rox1 cells and from 242.0 ± 5.9% to 140.7 ± 2.1% in Rox2 cells (Figure 5F). It is well known that Src is regulated by phosphorylation, the kinase activity of this enzyme is notably repressed by a phosphorylation on the tyrosine 527. We have therefore studied the phosphorylation status of Src in our cells transfected with a control siRNA or with siRNA directed against the ubiquitous calpains. Our results show that Src is less phosphorylated on the Y527 in our resistant cells than in the sensitive cells, suggesting that Src is more active in our Rox1 and Rox2 cells. The repression of calpain expression induces a strong phosphorylation of Src in our three sub-lines (Figure 5G).

These data show that Src mediate the activation of Nox1 by calpains in our resistant cells. The over-activated calpains maintain Src in its unphosphorylated active form, thus leading to the activation of Nox1 and to the increase of ROS production.

### Src and p38 MAPK kinases are involved in the resistance to oxaliplatin

**Figure 6:**
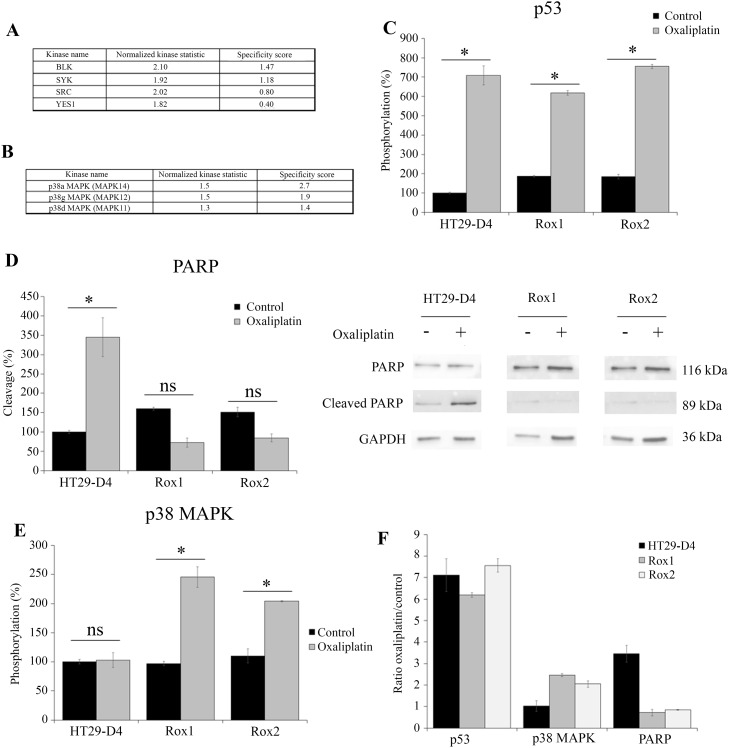
Comparison of HT29-D4, Rox1 and Rox2 signaling pathways HT29-D4 and Rox1 cells were seeded in 6-well plates and incubated in the absence or in the presence of 2 μM of oxaliplatin for 4 hours. The cells were lysed and 0.5 μg of proteins were used for Pamgene kinase activity assay. The data were analyzed using the Bionavigator software to compare the kinase activity of HT29-D4 and Rox1. The top kinase lists obtained in the absence of in the presence of oxaliplatin are presented in (**A** and **B)**, respectively. A positive normalized kinase statistic value indicate a kinase activity higher for Rox1 than for HT29-D4.HT29-D4, Rox1 and Rox2 cells were seeded in 6-well plates and were treated in the absence (Control) or in the presence of 100 μM of oxaliplatin (Oxaliplatin) for 24 hours. The cells were then lysed and 37.5 μg of proteins were used for the PathScan assay. The data were analyzed and the phosphorylation levels of p53 (**C**) and p38 (**E**) as well as the cleavage of PARP (**D**) were compared. The results obtained for PARP were also confirmed by Western blots using lysates of HT29-D4, Rox1 and Rox2 cells treated with or without 100 μM of oxaliplatin (D). The effects of oxaliplatin were visualized by calculating the ratio between the treated and untreated cells (**F**). Asteriks indicate a statistical significance with *p*<0.05.

To have a better understanding of the pathways involved in the resistance of our cells to oxaliplatin, we have performed a screening of the kinase activities of our sensitive and resistant cells, incubated in the absence or in the presence of oxaliplatin during 45 minutes and 4 hours. The kinase activity was measured using PamGene technology and the BioNavigator software. The data obtained in the absence of oxaliplatin clearly show that the activity of Src kinases is increased in the resistant cells in comparison to the sensitive cells (Figure 6A). Indeed, the kinases of the Src family were in the top kinase list with high specificity scores (1.47 for BLK, 0.80 for SRC and 0.40 for YES1) and high normalized kinase statistics (2.10, 2.02 and 1.82 for BLK, SRC and YES1, respectively). These data show that Src kinases are strongly activated in our resistant cells, confirming the results obtained by studying Src phosphorylation. The treatment of our cells with oxaliplatin for 4 hours induces a major and significant increase of p38 MAPK activity, as shown in Figure 6B. Indeed, the kinases of the p38 MAPK family were in the top list with high specificity scores (2.7 for p38α MAPK (MAPK 14), 1.9 for p38γ MAPK (MAPK12) and 1.4 for p38δ MAPK (MAPK 11)) and high normalized kinase statistics (1.5, 1.5 and 1.3 for p38α MAPK, p38γ MAPK and p38δ MAPK, respectively; Figure 6B). These results show that p38 MAPK are activated by oxaliplatin in our resistant cells. p38 MAPK is well known to be strongly implicated in cell survival, notably in response to chemotherapy.

To complete these data and observe the effects of oxaliplatin on the various signaling pathways, we performed a screening of signaling pathways using the Cell Signaling Technology PathScan (Figure 6, Supplementary Figures 7 and 8). Our results show major modifications in the activation of several signaling pathways. First of all, oxaliplatin was able to induce the phosphorylation of p53 in both the sensitive and the resistant cells, but failed to induce to cleavage of PARP in our resistant cells (Figure 6C-6D). Indeed, in the sensitive cells the cleavage of PARP was increased from 100% to 344.8 ± 3.7% by oxaliplatin. On the opposite, in Rox1 and Rox2 cells, the cleavage was reduced from 160.1 ±2.4% and 147.4 ± 5.0% to 72.5 ± 16.8% and 86.7 ± 0.9%, respectively. These data were confirmed by Western blots (Figure 6D). The cleavage of caspase 3 was too weak to be analyzed (data not shown). This study using the PathScan also confirmed the results obtained for p38 MAPK using Pamgene technology. Indeed, the oxaliplatin treatment induced a strong increase of p38 MAPK phosphorylation in both Rox1 and Rox2 cells, by more than 2 folds, while it had no effect in the sensitive cells (Figure 6E-6F). The levels of phosphorylation of the other signaling pathways measured by the PathScan were too weak to be analyzed (data not shown).

### p38 MAPK plays a major role in the resistance to oxaliplatin

**Figure 7:**
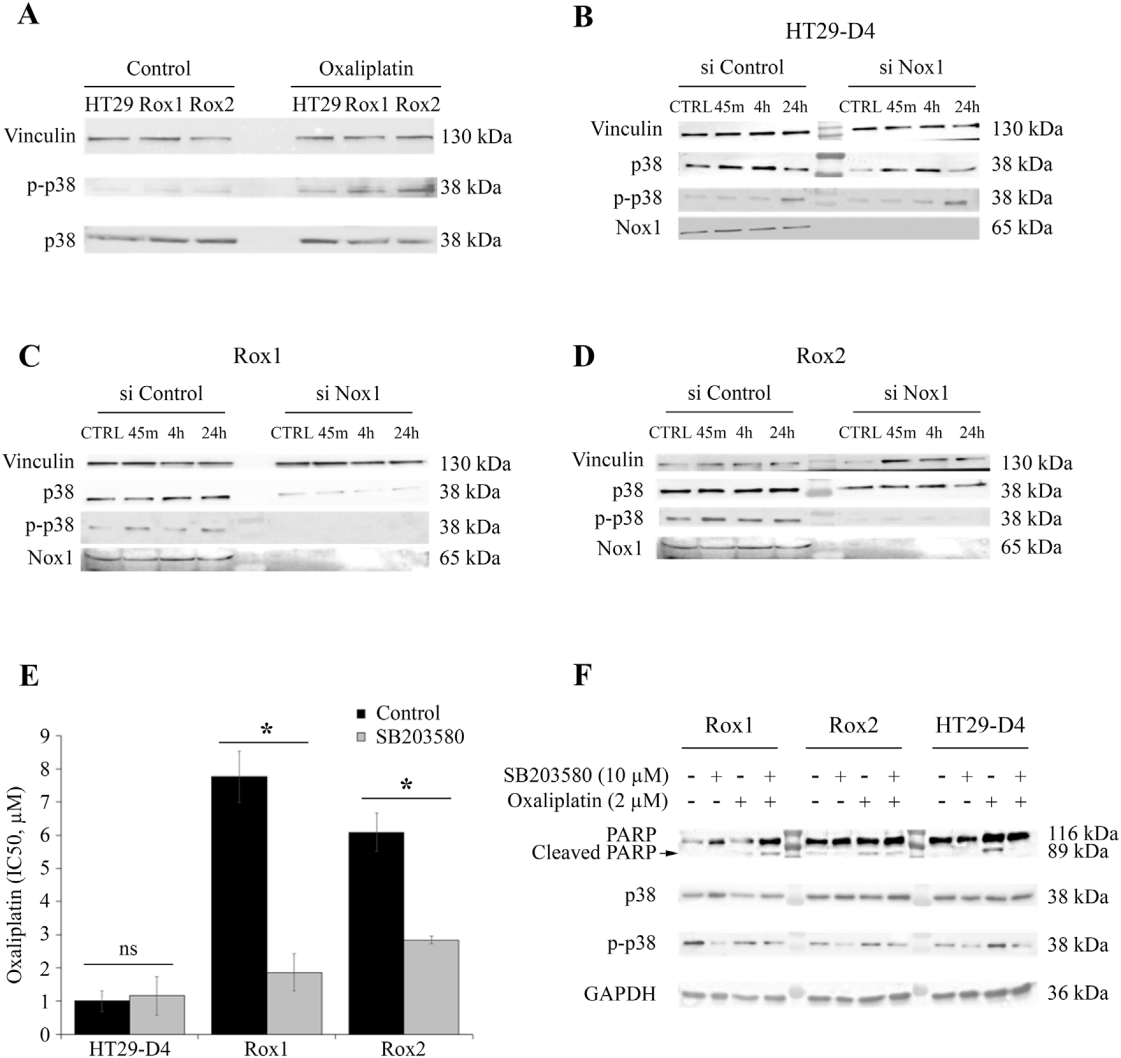
Implication of p38 in the resistance to oxaliplatin HT29-D4, Rox1 and Rox2 cells were seeded in 6-well plates and incubated in the absence (control) or in the presence of 2 μM of oxaliplatin. The cells were lysed and equal amounts of proteins were processed for immunoblotting using antibodies against vinculin, p38 and phospho-p38 (thr180/tyr 182) **(A)**. HT29-D4, Rox1 and Rox2 cells were transfected with control siRNA (si Control) and Nox1 specific siRNA (si Nox1). The cells were incubated in the absence (CTRL) or in the presence of 2 μM of oxaliplatin for 45 minutes (45m), 4 hours (4h), 24 hours (24h). The cells were lysed and equal amounts of proteins were processed for immunoblotting using antibodies against vinculin, p38 and phospho-p38 (thr180/tyr 182) **(B** to **D)**. Cytotoxicity assays were performed with HT29-D4, Rox1 and Rox2 treated with oxaliplatin and incubated in the absence (Control) or in the presence of SB203580, a specific inhibitor of p38 (5 μM) **(E)**. The cleavage of PARP was studied by Western blot performed with lysates from HT29-D4, Rox1 and Rox2 cells treated with or without oxaliplatin (2 μM) +/- SB203580 (10 μM) **(F)**. Asteriks indicate a statistical significance with *p*<0.05.

Our results obtained with PamGene technology and Cell Signaling PathScan suggest a potential implication of p38 MAPK pathway in the resistance of our cells to oxaliplatin. To confirm this involvement, we have studied the phosphorylation status of p38 MAPK in our sensitive and resistant cells. As shown in Figure 7A, p38 MAPK phosphorylation is weak in both our sensitive and resistant cells. However, oxaliplatin induces the phosphorylation of p38 MAPK in the resistant cells but not in the sensitive ones. These results confirm those obtained with the PathScan assay.

We have then studied the effects of the down-regulation of Nox1 expression on the phosphorylation of p38 MAPK. Our results show that the inhibition of Nox1 expression has a very limited effect on the phosphorylation of the p38 MAPK in the sensitive cells (Figure 7B). On the opposite, in Rox1 and Rox2 cells, the repression of Nox1 induces a very strong inhibition of the phosphorylation of p38 MAPK, even leading to the inhibition of p38 MAPK expression in Rox2 cells (Figure 7C-7D).

As p38 MAPK pathway activation is well known to be involved in cell survival, we have studied the effects of a specific p38 MAPK inhibitor on the cytotoxic effects of oxaliplatin on our three sub-lines. The IC_50_ of oxaliplatin for HT29-D4 cells was not modified by the inhibitor, increasing only from 1.0 ± 0.3 μM in the absence of SB203580 to 1.2 ± 0.6 μM in the presence of this inhibitor (Figure 7E). The involvement of p38 isoform in the resistance to oxaliplatin was confirmed using this inhibitor on resistant cells. Indeed, the repression of p38 activity induced a strong increase of oxaliplatin efficiency, the IC_50_ being reduced to 1.9 ± 0.6 μM for Rox1 and 2.8 ± 0.1 μM for Rox2 (*p*<0.05; Figure 7E).

To understand how p38 MAPK acts on the effects of oxaliplatin, we analyzed the induction of apoptosis in our cells by studying the cleavage of PARP. As shown in Figure 7F, oxaliplatin was able to induce the cleavage of PARP in our sensitive cells, while it failed to do so in the resistant ones. However, we observed a strong induction of the cleavage of PARP in our resistant cells when the cells are treated with oxaliplatin in combination with p38 inhibitor SB203580 (Figure 7F).

Taken together, these data show that the ROS produced by Nox1 in the resistant cells induced the activation of p38 MAPK leading to an inhibition of apoptosis and thus to cell survival.

## DISCUSSION

Resistance to chemotherapy is one of the major factors limiting the effectiveness of the treatments, particularly in colorectal cancer. Previous works have shown an important role of ROS (especially produced by Noxs and Duoxs) as they are necessary for the cytotoxicity of oxaliplatin [5, 17, 18] We showed here that the impact of ROS is also major in the context of colorectal cancer cells resistant to oxaliplatin. The inhibition of the production of ROS, particularly by Nox1, induced a further increase of the IC_50_ values in the resistant cells. These results confirm that, as in sensitive cells, ROS from Nox1 are involved in the cytotoxicity of oxaliplatin. Paradoxically, resistant cells exhibit a basal level of ROS produced by Nox1 almost twice as high as sensitive cells. However, the oxaliplatin treatment of the resistant cells leads to a reduction in the level of concomitant superoxide ion to an increase in the production of hydrogen peroxide inducing the activation of the survival pathways in the resistant cells. The activation of these survival pathways relies on the activation of p38 MAPK downstream of Nox1 in resistant cells. In agreement with this result, Shi et al. have shown that ROS can protect cells against oxaliplatin-induced cell death by activating autophagy [19].

The complexity of regulation of ROS, the diversity of species produced and the diversity of their cellular impact represents a limit to the use of modulators of oxidative stress for therapeutic purposes. To understand how to modulate oxidative stress to improve the effectiveness of anti-cancer therapies, numerous studies have focused on the role of superoxide and hydrogen peroxide. The intracellular concentration of H_2_O_2_ has been shown to be critical because it reaches the toxicity threshold in tumor cells. Increasing the level of H_2_O_2_ for normal cells tends to stimulate proliferation whereas in cancer cells it slows down tumor proliferation and leads to the death of an apoptotic cell [20]. Conversely, any agent that decreases intracellular levels of H_2_O_2_ improves tumor growth [21]. Moreover, in tumor cells, it appears that a predominant increase of superoxide favors cell survival and oncogenesis whereas an inclination in favor of hydrogen peroxide prevents carcinogenesis by facilitating the signaling of cell death [22]. Our results on sensitive colorectal cancer cells are consistent with the mechanism previously described. The production of superoxide and hydrogen peroxide induced by oxaliplatin treatment promotes the cytotoxicity of oxaliplatin and depends mainly on the activation of Nox1 (Figure 2E and 2F). Furthermore, the increase in superoxide ion production in basal cell resistant cells is associated with a greater proliferation of cells on 3D culture models (Figure 1C). Our data add a new piece to the puzzle by showing that the transient increase of ROS production observed in sensitive cells leads to the activation of apoptosis, while superoxide ions will be extremely rapidly transmuted into peroxide in resistant cells, leading to cell survival. This study shows once again the double and antinomic role played by ROS in cancer and highlights the very fine limit of regulation existing in these cells [23, 24]. These data are shown for an oxaliplatin concentration of 2 μM (toxic for sensitive cells and poor effect on resistant cells), which represents the plasma concentration in the treated patients. It should be noted that we have observed this dismutation like effect on sensitive cells at oxaliplatin concentrations of 100 μM but in this situation the shift of superoxide to hydrogen peroxide induced cell death [5]. Thus, this phenomenon would be triggered at doses much higher than the plasma concentrations of oxaliplatin in the sensitive cells compared to the resistant cells. Finally, in our model, the production of H_2_O_2_ in our cells returned to the basal level at 24h of treatment and therefore does not seem compatible with an increase in Duox2 expression as observed in a recent study [17].

The level of expression of Nox1 and some of its regulatory partners is known to be increased in colorectal cancer and could explain the increase in Nox1 activity observed in our resistant cells [25, 26]. In our study, we did not observe any increase in the expression of Nox1 or NoxO1 between resistant or sensitive cells under treatment with oxaliplatin. However, we observed a significant increase of NoxA1 expression in our resistant cells consistent with the observed increase in Nox1 basal ROS production.

In order to identify the signaling pathways involved in the resistance to oxaliplatin, we carried out kinomic screening using PamGene array and PathScan signaling array. Our data obtained using PamGene show an increase in Src kinase and p38 MAPK activation in the resistant cells, in comparison to the sensitive ones. Consistent with our result, p38 MAPK is strongly involved in cell survival and linked to the resistance to irinotecan and 5-FU in colorectal cancer cells [27, 28]. Our data also show an absence of activation of the effectors of apoptosis in our resistant cells after treatment with oxaliplatin. There is indeed a decrease in the cleavage of PARP and an absence of cleavage of the caspase 3. There would therefore be a dysfunction at the level of one of the effectors of the apoptosis [29]. We also observed an increase of PRAS40 phosphorylation in our sensitive cells in the presence of oxaliplatin, while the phosphorylation of this protein was decreased in our resistant cells. This result is surprising as it was shown that a decrease of PRAS40 phosphorylation can increase apoptosis and reduce tumor development [30, 31]. However, it has been shown that the decrease of PRAS40 phosphorylation can also prevent apoptosis in HeLa cells through Akt and/or Pim1 activation [30]. Finally, we confirmed with the PathScan array that p38 MAPK activity was increased in resistant cells compared to sensitive cells. Several publications have highlighted the connection existing between the ROS produced by Nox1 and the activation of p38 MAPK [32]. We have observed that p38 MAPK was strongly phosphorylated and thus activated in our resistant cells. This activation of p38 MAPK is required for the resistance of cells to oxaliplatin as the use of a p38 MAPK inhibitor (SB203580;10 μM) strongly reduces the IC_50_ of oxaliplatin for these cells. Using siRNA, we could show that the ROS produced by Nox1 are required for the activation of p38 MAPK in the resistant cells.

Previous works have highlighted that calpains have also antinomic roles in the response of cancer cells to chemotherapies. Indeed, it was shown that calpains are involved in both the cytotoxic effects induced by chemotherapy and the resistance to the treatments, depending on the type of cancer. In colorectal cancer, a recent study has shown that calpain 2 is involved in the resistance of cancer cells to irinotecan [16]. To have a better understanding of the roles played by calpains in the resistance to oxaliplatin, we have compared the expression and activity of the ubiquitous calpains between our sensitive and resistant cells. Our data show that the expression levels of calpain 1 and calpain 2 are not modified but that their activity is strongly increased. Our results also show that both calpains are responsible for this activity increase. This calpain over-activation in our resistant cells can be surprising as several publications have shown that chemotherapy molecules can induce apoptosis by activating calpains via short-term induction of calcium influx [15, 33]. Our data show that the inhibition of calpains in resistant cells induces a significant increase in their resistance. We can thus hypothesize that oxaliplatin activates calpains to induce the apoptosis of our resistant cells, but that dysfunctions in the apoptotic pathways lead to cell survival [29]. To complete these data, we have studied the different pathways that could be responsible for this calpain over-activation in our resistant cells. It is well known that calpains are activated by calcium and by ERK/MAPK pathway but it was also shown that ROS can regulate calpain activity [8, 9, 34, 35]. Our results exclude the hypothesis of a stimulation of calpains due to the over-production of ROS, as the inhibition of Nox1 by DPI had no effect on calpain activity. Similarly, the inhibition of MEK1 using PD98059 induced no change in the activity of calpains.

Our data support the hypothesis of an activation of calpains by calcium influxes. Indeed, we have observed a significant increase of the intracellular concentration of calcium in our resistant cells in comparison to the sensitive ones. We were also able to show for the first time that PKC delta regulates negatively calpain activity. Indeed, an inhibition of PKC delta leads to a significant increase in calpain activity. However, complementary studies will be required to know if this regulation has a role in the development of the resistance to oxaliplatin and how this regulation is mediated.

Our data exclude a regulation of calpain activity by Nox1-produced ROS in our cells, however it is possible that calpains regulate Nox1 activity. Several studies have highlighted the existence of regulatory links between calpains and NADPH oxidases, however these regulations are different depending on the cell types studied. As said before it was proved that ROS can regulate calpain activity [34, 35], but the opposite was also shown. Chen and colleagues have shown that the inhibition of calpains results in reduction of ROS [36]. In addition, some agents increase calpain activity via calcium, resulting in an inhibition of ROS production [37]. Silibinin was also shown to induce an increase of calpain activity leading to an increase of ROS production [38]. Our experimental data show that the repression of calpain expression, and therefore of calpain activity, leads to a strong inhibition of the production of ROS in our resistant cells. However, in our sensitive cells the inhibition of calpain 1 or both calpains 1 and 2 induced an increase of ROS production. These results show a positive regulation of Nox1 by the ubiquitous calpains in the resistant cells, while calpains are negatively regulating Nox1 in the sensitive HT29-D4 cells. We are thus showing for the first time an inversion of regulation of Nox1 by calpains according to the resistance status of our cancer cells. These observations are confirmed by the results that we obtained when inhibiting PKC delta expression. Indeed, the repression of PKC delta expression induced an increase of calpain activity in both our sensitive and resistant cells, leading to an increase of superoxide production in sensitive cells and to a decrease of this production in our resistant cells.

In order to have a better understanding of this inversion of regulation, we have studied and characterized both the inhibition of Nox1 by calpains in the sensitive cells and the activation observed in the resistant ones. Concerning the inhibition observed in the sensitive cells, we hypothesized that calpains could cleave one or several components of Nox1 complex. Nox1 requires NoxA1, NoxO1 and Rac1 to be active. None of these proteins are known as calpain substrates. Tiam1, a Rac1 activator, was shown to be cleaved by calpains in fibroblasts [39], however no cleavage was observed in our cells (data not shown). Using a cleavage prediction program (CalCleave;calpain.org), we observed that only NoxA1 could be cleaved by calpains. This prediction correlates with our data. Indeed, we observed that oxaliplatin induced a disappearance of NoxA1 in our cells, however this depletion was partially inhibited when calpain expression was repressed. The depletion of NoxA1 could thus be the result of a degradation mediated by calpains. The incubation of immunoprecipitated NoxA1 with purified calpain 1 led to the appearance of a cleaved NoxA1 band, confirming the existence of one site of cleavage. The cleaved NoxA1 being around 49kDa, the cleavage site is located at one extremity of the protein. Using an immunoprecipitated DDK-tagged NoxA1 we could show that the cleavage is occurring at the C-terminus of NoxA1. This cleavage is thus occurring in a SH3 domain, known to be involved in the binding of NoxA1 to NoxO1 required for the activation of Nox1 (Supplementary Figure 5). It is the first time that NoxA1 is identified as a calpain substrate. This cleavage explains how calpains regulate negatively Nox1 activity in our sensitive cells. In these cells, oxaliplatin induces a strong increase of ROS production by activating Nox1. Calpains, also activated by oxaliplatin, cleave NoxA1, thus inhibiting Nox1 and reducing ROS production to a regular level, as observed after a 24-hour treatment with oxaliplatin. This short-term peak of ROS production leads to cell apoptosis. In these cells, calpains and Nox1 are both involved in the cytotoxic effects of oxaliplatin.

In our resistant cells, the oxaliplatin-induced cleavage of NoxA1 by calpains is present however the increase of expression of NoxA1(Figure 2C, Supplementary Figure 3C) in this cells reduce the efficiency of this ROS production inhibition compared to sensitive cells. This difference could be due to different locations of calpains and NoxA1. Src kinase is an excellent candidate to explain this regulation. It is known that Src can phosphorylate NoxA1 on tyrosine 110 in HT29 cells, thus increasing the production of ROS [40]. It was also previously shown that calpain 2 can induce the activation of Src kinase by cleaving PTP1B [41]. Src kinase activity is inhibited by a phosphorylation on tyrosine 527. PTP1B is a phosphatase, activated by calpain cleavage, known to dephosphorylate the tyrosine 527 of Src, leading to the kinase activation. We can thus hypothesize that calpain could induce Nox1 activation by derepressing Src activity. As we showed that Src activity is decreased in resistant cell using the Pamgene array, we confirmed that hypothesis by studying Src phosphorylation and by using Src inhibitor (Figure 5F-5G). Indeed, we have observed that Src is less phosphorylated and thus more active in our resistant cells compared to sensitive cells. Moreover, the inhibition of Src in these cells strongly reduced the production of ROS, while it has no effect in the sensitive cells. The implication of calpains in this phenomenon was confirmed using siRNA. The repression of ubiquitous calpain expression led to the phosphorylation of Src, and thus to its inactivation. Taken together our data clearly show that calpains inhibit Nox1 in the sensitive cells by cleaving NoxA1, while they activate Nox1 activity by activating Src kinase.

**Figure 8:**
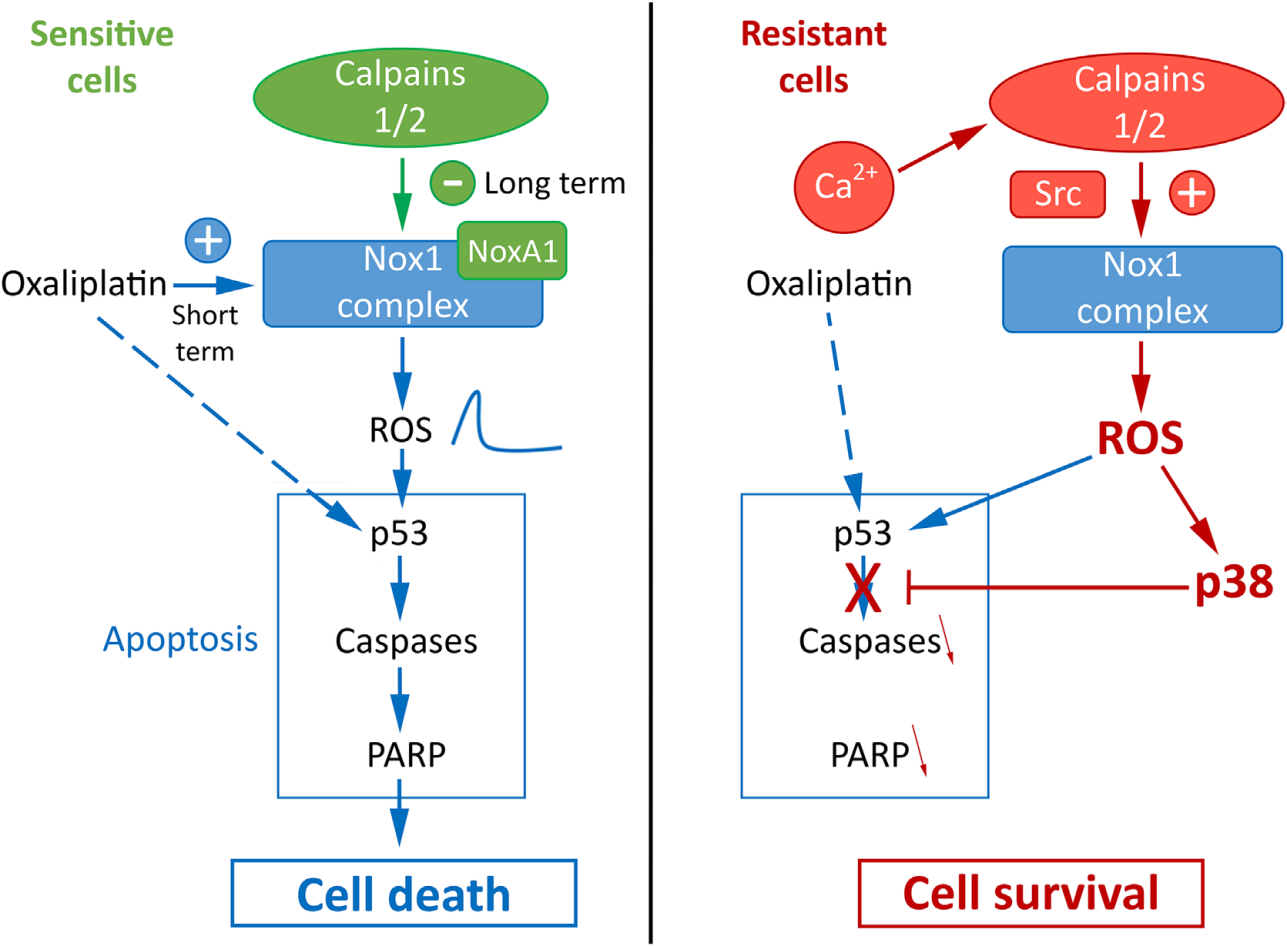
Proposed model for the regulation of oxaliplatin effects by calpains, Nox1 and p38 in sensitive and resistant colorectal cancer cells

Taken together, our results allow us to have a better understanding of the mechanism of resistance of cancer cells to oxaliplatin and to propose a new model presented in Figure 8. We could identify a new calpain/Nox1 pathway, regulated differently according to the resistance status of the cells. We have also identified NoxA1 as a new calpain substrate. In sensitive cells oxaliplatin induces the activation of Nox1 and thus the production of ROS. The activation of calpains leads to the cleavage and degradation of NoxA1, reducing Nox1 activity and returning ROS production to regular levels. The short-term peak of ROS induces the activation of p53, PARP and caspase 3 leading to cell death. It is important to note that p53 mutation status seems to have no impact on the resistance of our cell lines to oxaliplatin. Indeed we obtained similar results using HT29-D4 and RKO cells while these cells present a different p53 mutation status (R273H mutation in HT29-D4 cells, wild-type p53 in RKO cells). In the resistant cells, the strong calcium concentration induces calpain activation, leading to Src and thus Nox1 activation. The strong ROS production, maintained at high levels, induces the activation of p38 MAPK and thus cell survival. Our data also show that ROS are involved in both cell survival and cell death, per the level of their production. If the production is too low, oxaliplatin is unable to activate cell death, however if ROS production is too high, it leads to the activation of survival pathways through p38 MAPK activation. As p38 MAPK inhibitor are now under clinical evaluation for colorectal cancer treatment, it is interesting to consider that a strategy leading to maximize Nox1 activity associated to the inhibition of p38 MAPK would be beneficial for patients, particularly those resistant for oxaliplatin treatment.

## MATERIALS AND METHODS

### Tumor cell lines and culture conditions

Two human colon carcinoma cell lines, HT29-D4 and RKO, were routinely maintained in Dulbecco’s modified Eagle’s medium (DMEM) containing 10% fetal bovine serum (FBS) (GIBCO Cell Culture systems, Invitrogen), supplemented with 2 mM L-glutamine, at 37°C in a humidified atmosphere with 5% CO_2_. HT29-D4 cell line was originally derived from HT29 colon adenocarcinoma cell line [42].

### Reagents and antibodies

#### Most of the reagents were supplied by Sigma-Aldrich.

The following reagents were used: oxaliplatin (L-OHP) stored at 5.4 mg/mL (12.5 mM) and used at different concentrations; apocynin, inhibitor of ROS production [43], stored at 100 mM and used at 0.5 mM (concentration validated by previous works); DPI (Merck Millipore) inhibitor of ROS production, stored at 10 mM and used at 10 μM (concentration validated by previous works); the ML171 (Merck Millipore), a Nox1 specific inhibitor, stored at 10 mM and used at 1.5 μM (in agreement with the IC_50_ values provided by the manufacturer); the MDL28170 (Calbiochem, Merck Millipore), a specific calpain inhibitor, stored at 200 mM and used at 2.5 μM; the PD98059 (Calbiochem, Merck Millipore), a specific MEK1 inhibitor, the SB203580, a specific p38 MAPK inhibitor, stored at 10 mM and used at 10 μM. The following primary antibodies were used: anti-calpain1 (diluted at 1/1,000, ref. 2556, Cell Signaling Technology); anti-calpain 2 (diluted at 1/1,000, ref. 2539, Cell Signaling Technology); anti-DDK tag (FLAG) (diluted at 1/ 1,000, ref. TA50011, Origene Technologies); anti-GAPDH (1/20,000, ref. G8795); anti-Nox1 (1/1,000, ref. ab121009, Abcam); anti-NoxA1 (1/1,000, ref. ab68523, Abcam); for NoxA1 immunoprecipitation: anti-NoxA1 (ref. H00010811-PW1, Abnova); anti-NoxO1 (1/1,000, ref. ab34761, Abcam); anti-p38 MAPK (1/1,000, ref. sc-535, Santa Cruz); anti-phospho-p38 MAPK (Thr180/Tyr182) (1/500, ref. sc17852, Santa Cruz); anti-PARP and anti-cleaved PARP (1/1,000, ref. 9542 and 9541, respectively, Cell Signaling Technology); anti-vinculin (1/20,000, ref. V9264). The HRP-coupled secondary antibodies were purchased from Cell Signaling Technology.

### Selection of oxaliplatin-resistant cell lines

Resistance to oxaliplatin (L-OHP) was induced by exposing the sensitive cells to increasing concentrations of the drug. The initial dose was 0.01 μg/mL and the final concentration, 0.87 μg/mL (2 μM), corresponds to the clinically relevant plasma concentration of oxaliplatin [44]. The oxaliplatin concentration was increased every two passages in two different ways: rapidly (for Rox1 cells) and slowly (for Rox2 cells). For Rox1 cells, the concentration was doubled, while it was increased by 0.1 μg/mL (0.25 μM) for Rox2. Once selected, the resistant cells were grown in the regular culture medium supplemented with 0.87 μg/mL oxaliplatin. For both HT29-D4 and RKO cell lines two groups were considered for investigations: the parental cells and the chemoresistant cells (HT29-D4-Rox1 and 2, and RKO-Rox1 and 2).

### Cytotoxicity assay

Cell viability was determined using the MTT assay. This assay is based on the ability of mitochondrial dehydrogenase enzyme to convert the yellow water-soluble tetrazolium 3-(4,5-dimethyl-thiazolyl)-2,5-diphenyl-tetrazolium bromide (MTT) into violet formazan compound, whose absorbance is proportional to the amount of living cells. After counting and plating the sensitive or resistant (Rox) cells (50,000 cells/mL) in 96-well plates with DMEM culture medium, the cells were exposed to increasing concentrations of the drug (from 0 to 100 μM) for 72 hours. The culture medium was then replaced with DMEM containing 0.5 mg/mL MTT. After a 2-hour incubation, the cells were lysed and the formazan solubized using pure DMSO. The optical density (OD) was measured at 600 nm in a plate reader (Multiskan RC, Labsystems). The data were expressed as percentage of survival (using the untreated cells as 100%) and subjected to statistical analysis (*n* = 5). The IC_50_ were determined using the Chou and Talalay method [45].

### 3D MTT assay

After counting, the cells were seeded on a 96-well plate with round bottom, at a density of 1,000 cells per well in a medium containing 20% methylcellulose (6 g/L). After a 72-hour incubation allowing the spheroid formation, the cells were treated with increasing concentrations of oxaliplatin (from 0.25 μM to 100 μM). The treatment was renewed every 72 hours during 15 days. The medium was then removed and cells were incubated in culture medium containing 0.5 mg/mL MTT for 24 hours (time required for the total coloring of the spheroid). Medium containing MTT was then removed and the cells were lysed with pure DMSO. The optical density was measured at 600 nm using a plate reader (Multiskan RC, Labsystems). The IC_50_ were determined by the method of Chou and Talalay [45]. In addition, pictures of cells were taken every day to follow the spheroid evolution. Their areas were calculated using the NIH ImageJ software.

### Preparation of cells extracts

The cells were washed in ice-cold PBS (phosphate buffered saline) and lysed in hypotonic lysis buffer (Tris buffered saline (TBS) pH 7.5, 0.1% Sodium dodecyl sulfate (SDS), 1 mM EDTA, 1% Triton X-100; cocktails of protease and phosphatase inhibitors (Halt phosphatase and Halt protease inhibitor kits, Thermo Fisher Scientific). Lysates were centrifuged at 11,300 g for 10 minutes at 4 °C to remove cell debris. A protein quantification assay was then performed using the Protein Assay Dye Reagent Concentrate (Bio-Rad). Loading buffer (Laemmli sample buffer, 62.5 mM Tris-HCl pH 6.8, 25% glycerol, 2% (SDS); bromophenol blue, 350 mM dithiothreitol (DTT)) was added to the proteins and the samples were denatured at 95°C for 5 minutes.

### Western blotting

Protein samples were loaded (30 μg/lane) and separated on 10% sodium dodecyl sulfate polyacrylamide gels. The separated proteins were electrophoretically transferred on Nitrocellulose Blotting Membrane (Amersham Protan, GE Healthcare) using a transfer system (Bio-Rad). The membranes were incubated with blocking solution (5% nonfat milk) for 1 hour and then incubated overnight with the proper primary antibodies. The membranes were then washed three times with a PBST solution (PBS plus 0.05% Tween20) and incubated with horseradish-peroxidase-conjugated secondary antibodies for 1 hour. The membranes were again washed three times with PBST, and revealed using chemiluminescence HRP substrate (Merck Millipore) and the G-Box (Syngene). The band intensities were quantified using the NIH ImageJ software.

### Calpain activity assay

The cells were seeded on a black bottom 96-well plate (20,000 cells per well). After 24 hours of culture, the cells were incubated with different treatments according to the experiment protocol. The cells were then incubated with 25 mM of t-boc-LM-CMAC, a fluorogenic calpain substrate provided by Invitrogen (Life Technologies). After a 25-minute incubation, the cells were washed with PBS and the fluorescence was quantified using a Fluoroskan (FL Fluoroskan Ascent, Labsystems; excitation wavelength: 355 nm, emission wavelength: 460 nm). The cells were then fixed in 1% glutaraldehyde for 10 minutes and stained with crystal violet (0.1%) for 30 minutes. After several washes with PBS, cells were lysed in pure DMSO and the optical densities were measured using a plate reader (Multiskan RC, Labsystems). The results obtained with the t-boc-LM-CMAC were normalized using the crystal violet OD values. They were then compared to the control condition and expressed as a percentage.

### Measurement of superoxide production

The cells were seeded on a white 96-well plate (20,000 cells per well). After 24 hours of culture, the cells were incubated with the different treatments according to the experiment protocol. The cells were then incubated with 1 mM of NADPH (cofactor of the NADPH oxidases) and 10 μM of lucigenin. The superoxide production was calculated by integrating the luminescence values measured every minute during a 45-minute period using a Fluoroskan plate reader (FL Fluoroskan Ascent, Labsystems). The cells were fixed with glutaraldehyde (1%) for 10 minutes and stained with crystal violet (0.1%) for 30 minutes. After several washes with PBS, cells were lysed in pure DMSO and the optical densities were measured using a plate reader (Multiskan RC, Labsystems). The results obtained with lucigenin (in RLU) were normalized using the crystal violet OD values. They were then compared to the control condition and expressed as a percentage.

### Measurement of intracellular H_2_O_2_

The H_2_O_2_ generation was measured by dichlorodihydrofluorescein diacetate (H2-DCFDA). After seeding of the cells in black 96-well plates (20,000 cells per well) and incubation for the desired time with the different treatments. The culture media were replaced by measurement buffer containing 10 μM of H2-DCFDA for 30 min. Cells were then washed with measurement buffer without H2-DCFDA, and fluorescence was measured at 37°C using the Fluoroskan Ascent FL fluorimeter (excitation: 490 nm, emission: 538 nm; Labsystems, France). The H_2_O_2_ production was calculated by integrating the fluorescence values measured every minute during a 1-hour period. The results obtained with DCFDA (in RFU) were normalized using the crystal violet OD values (for crystal violet staining see description above). They were then compared to the control condition and expressed as a percentage.

### Measurement of extracellular H_2_O_2_

The cells were seeded on a black 96-well plate (20,000 cells per well). After 24 hours of culture, the cells were incubated with the different treatments according to the experiment. The cells were then treated with 50 μM Amplex Red reagent and 0.1 U/mL HRP (Amplex Red Hydrogen Peroxide/peroxidase Assay Kit, Invitrogen, USA) and were incubated at room temperature for 30 minutes protected from the light. The fluorescence was measured at 37°C using POLARstar Omega (excitation: 560 nm, emission: 590 nm; BMG LabTech, Germany). The results were normalized using the crystal violet OD values, compared to the control condition and expressed as a percentage.

### Cell transfection

HT29-D4 sensitive and resistant cells were transfected with siRNAs directed against calpain 1 and/or 2 and against Nox1 (a scramble siRNA was used as a control). The 22-nucleotide long siRNA used in these experiments were purchased from Qiagen (siRNA calpain 1 target sequence: 5’-TAGGATCATCAGAAACACAA-3’; siRNA calpain 2 target sequence: 5’-CTCGGAGGCCATCACGTTTCA-3’; siRNA Nox1: target sequence not provided by the supplier). Transfections were performed by electroporation using the Nucleofector Technology (Lonza). Different experimental procedures were compared to optimize the transfection protocol used (3 million cells, 100 μL of Nucleofector T, 300 nM siRNA, W-017 program). After electroporation, the cells were seeded in complete culture medium and incubated for 24 hours. The cells were then used in the different experiments. A part of the cells was seeded separately to monitor the transfection efficiency by Western blot.

### Immunoprecipitation and *in vitro* calpain degradation

NoxA1 immunoprecipitation was performed on HT29-D4 cells, transfected or not with the plasmid containing NoxA1-DDK gene. The cells were washed with cold PBS and incubated in RIPA buffer (Tris-HCl 50 mM, NP-40 1%, 0.5% sodium deoxycholate, 0.1% SDS, 150 mM NaCl, 2 mM EDTA, protease and phosphatase inhibitors (Halt protease and Halt phosphatase inhibitor cocktails, Thermo Fisher Scientific)) for 30 minutes at 4°C. After scrapping, the samples were sonicated for 20 seconds. Centrifugation was then carried out at 10,000 g for 10 minutes at 4°C. The lysates were incubated overnight at 4°C with 0.5 μg of primary antibody directed against NoxA1 (Abnova). The next day, 60 μg of protein A-sepharose beads (Roche) were added to the lysates and incubated for 1 hour at 4°C. The lysate-antibody-bead mixtures were centrifuged at 200 g for 5 minutes at 4°C and the beads were washed with RIPA buffer twice. To achieve *in vitro* calpain degradation, the beads were resuspended in a calcium buffer (50 mM HEPES pH 7.0, 50 mM NaCl, 1 mM CaCl_2_, 1 mM DTT). Five micrograms of purified calpain 1 (Calbiochem, Merck Millipore) were then added to the samples. Control samples without purified calpain were also prepared. The samples were incubated at 37°C for an hour, with regular stirring. After addition of Laemmli sample buffer, the samples were boiled at 95°C for 5 minutes and centrifuged at 11,300 g for 10 minutes. The supernatants were then used to perform Western blots.

### Measurement of cytosolic calcium variation

The cells, treated or not, were incubated in the Fura-2AM loading solution consisted of standard extracellular saline (SES; 10 mM HEPES pH 7.4, 135 mM NaCl, 5mM KCl, 1.2 mM MgCl_2_, 2 mM CaCl_2_, 1 mM bicarbonate and 5 mM glucose) with 0.1% BSA and 10 μM Fura-2AM (Thermo Fischer Scientific) at 37°C for 30 min. The loading solution was then removed and the cells were equilibrated in fresh SES buffer for 15 min and detached using trypsin. The fluorescence of the cell suspensions (1 mL) was recorded using a SFM 25 (Kontron Instruments; excitation wavelengths: 340 and 380 nm, emission wavelength: 510 nm). The changes in the intracellular calcium concentration were monitored using the Fura-2 340/380 fluorescence ratio.

### Intracellular signaling array

The PathScan Intracellular signaling array kit from Cell Signaling Technology was used to investigate the modification of the signaling pathway activation. After a 24-hour incubation in the presence or in the absence of oxaliplatin, the cells were lysed with the lysis buffer supplied in the kit complemented with protease and phosphatase inhibitors (Halt phosphatase and Halt protease inhibitor cocktails, Thermo Fisher Scientific). The lysates were centrifuged at 2,000 g for 3 minutes at 4°C. The supernatant was removed and the proteins were quantified using the Precision Red Advanced Protein Assay from Cytoskeleton, Inc.. The samples were diluted in lysis buffer to obtain a final concentration of 1.2 mg/mL in 50 μL. The slides were saturated for 15 minutes with a saturation buffer provided in the kit and 50 μL of the lysates were added to the different wells. After an overnight incubation at 4°C, the wells were washed during 5 minutes three times. The slides were incubated with the detection antibody provided in the kit for 1 hour at room temperature. After washing, the slides were treated with HRP-coupled streptavidin (provided in kit) for 30 minutes at room temperature. After another series of washes, the chemiluminescence was revealed using the Lumiglo/peroxide detection kit (provided by the manufacturer) and observed with the Syngene G-Box. The intensity of the chemiluminescence was quantified with NIH ImageJ software. After subtraction of the intensity of the negative control, the results were expressed as percentage using the positive control as 100%.

### Kinase activity assay

To study the kinase activity, the Protein Tyrosine Kinase (PTK) and the Serine-Threonine Kinase (STK) assays from PamGene were used. The HT29-D4 or HT29-D4-Rox1 cells were treated with oxaliplatin for different incubation times, collected and lysed using the M-PER lysis buffer (Thermo Fisher Scientific). A protein quantification assay was then performed using the Protein Assay Dye Reagent Concentrate (Bio-Rad). 5 μg and 1 μg were loaded on Protein Tyrosine Kinase and Serine-Threonine Kinase Pamchips, respectively. The phosphorylation of PamChip peptides were monitored by the PamStation^®^ 12, following the provided protocols (PamGene). The images were quantified using BioNavigator software (PamGene).

### Statistical analysis

For kinomic analysis, image analysis and signal quantification were performed using the BioNavigator^®^ software (PamGene). Peptides that showed kinetics (increase in signal intensity in time) were preselected (“QC list”). For each peptide, the comparisons between sensitive and resistant cells were performed using ANOVA. Kinexus Kinase Predictor was used to determine putative upstream kinases.

The Student’s *t*-test was used to compare the means and to determine whether the differences observed in our experiments were significant or not. All data were made in triplicate and were repeated at least three times except for the PathScan array. The difference is considered to be significant when the *p* value is less than 0.05 (significativity greater than 95%). In our figures, the values represent the mean plus or minus the standard deviation and the significance is represented by an asterisk (^*^).

## SUPPLEMENTARY MATERIALS FIGURES


