# Correction: Reversion of resistance to oxaliplatin by inhibition of p38 MAPK in colorectal cancer cell lines: involvement of the calpain / Nox1 pathway

**DOI:** 10.18632/oncotarget.25605

**Published:** 2018-06-01

**Authors:** Mathieu Chocry, Ludovic Leloup, Hervé Kovacic

**Affiliations:** ^1^ Aix-Marseille Université, INSERM, CRO2 UMR_S 911, Marseille 13385, France

**This article has been corrected:** The correct figures are given below:

The authors declare that these corrections do not change the results or conclusions of this paper.

**Figure 2:**
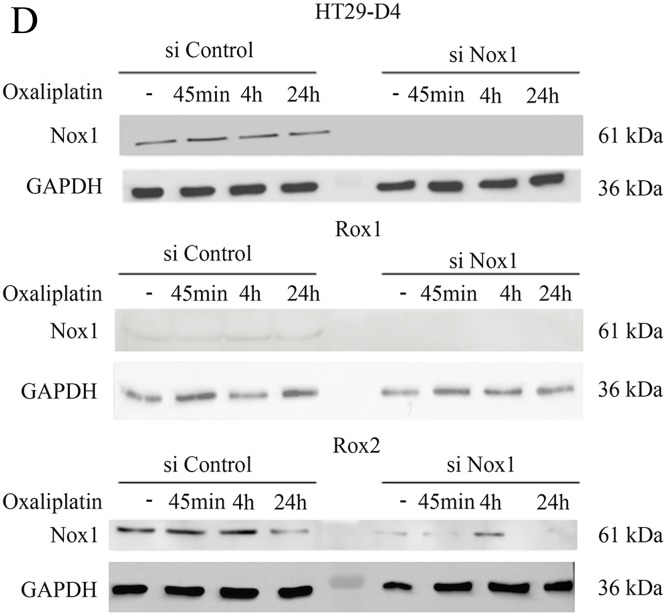
Implication of Nox1 in oxaliplatin-induced ROS production and cytotoxicity **(D).** Transfected cells were also seeded in white 96-well plates to perform lucigenin assays.

**Figure 3:**
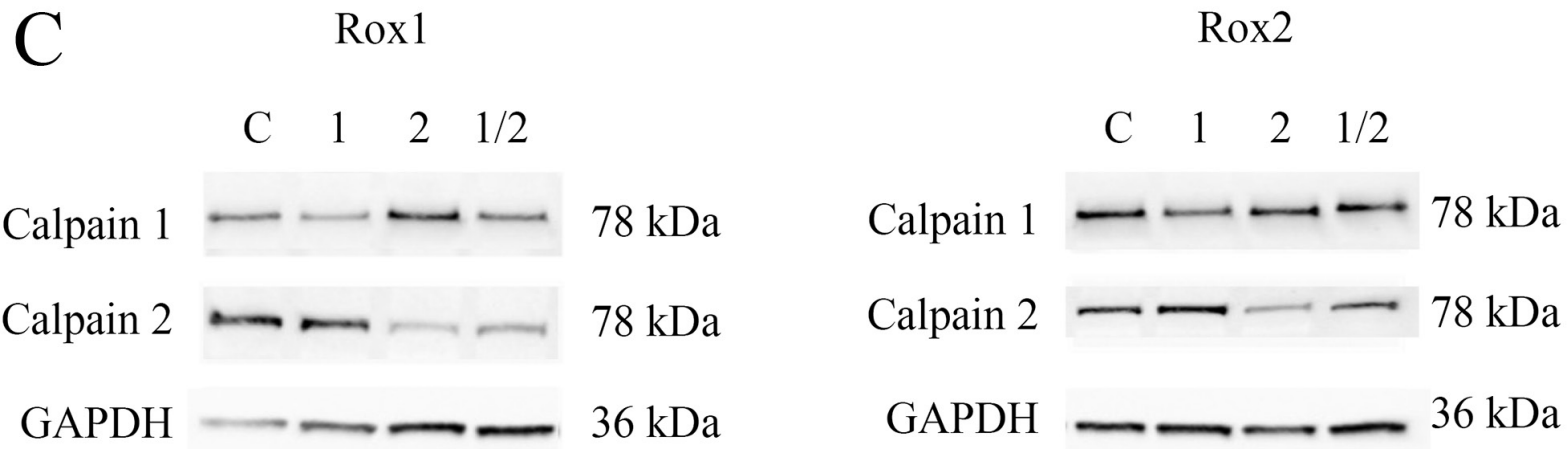
Study of calpain expression, activity and implication in oxaliplatin-induced cytotoxicity **(C).** The transfected cells were also seeded to perform 72-hour cytotoxicity assays **(C).** Asteriks indicate a statistical significance with *p*<0.05.

**Figure 7:**
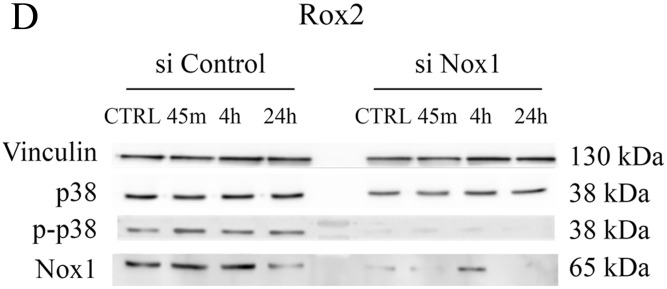
Implication of p38 in the resistance to oxaliplatin **(B to D).** Cytotoxicity assays were performed with HT29-D4, Rox1 and Rox2 treated with oxaliplatin and incubated in the absence (Control) or in the presence of SB203580, a specific inhibitor of p38 (5 μM).

Original article: Oncotarget. 2017; 8:103710-103730. https://doi.org/10.18632/oncotarget.21780

