# Supplementary material for: Reversion of resistance to oxaliplatin by inhibition of p38 MAPK in colorectal cancer cell lines: involvement of the calpain / Nox1 pathway
Source: Oncotarget. 2017 Oct 10;8(61):103710–30. doi: 10.18632/oncotarget.21780 (PMC5732761; doi:10.18632/oncotarget.21780)
Supplement: Supplementary file 1 [file oncotarget-08-103710-s001.pdf]

Reversion of resistance to oxaliplatin by inhibition of p38 MAPK in colorectal cancer cell lines: Involvement of the calpain / Nox1 pathway

SUPPLEMENTARY MATERIALS

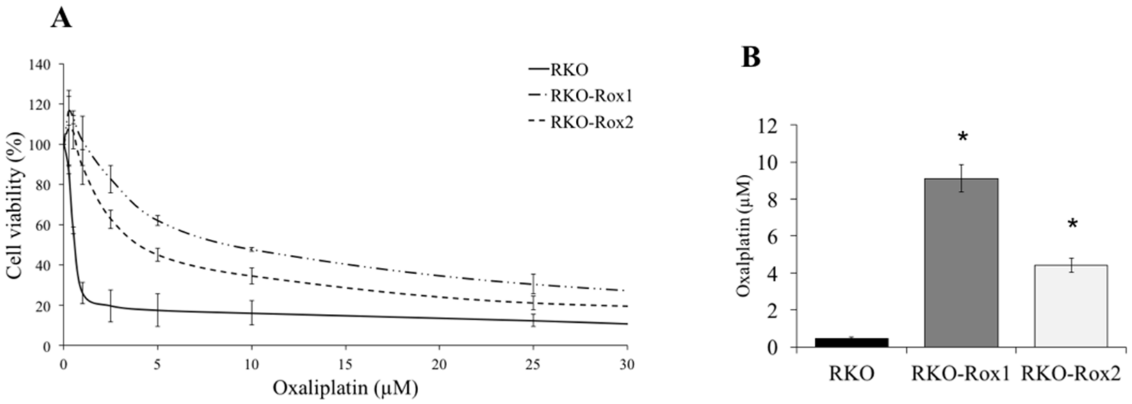

Supplementary Figure 1: Determination of the resistance status of selected cells. RKO, RKO-Rox1 (Rox1) and RKO-Rox2 (Rox2) were submitted to 72-hour cytotoxicity assays to oxaliplatin **(A)**. The IC50 were calculated using the Chou and Talalay's method **(B)**. Asteriks indicate a statistical significance with $p<0.05$.

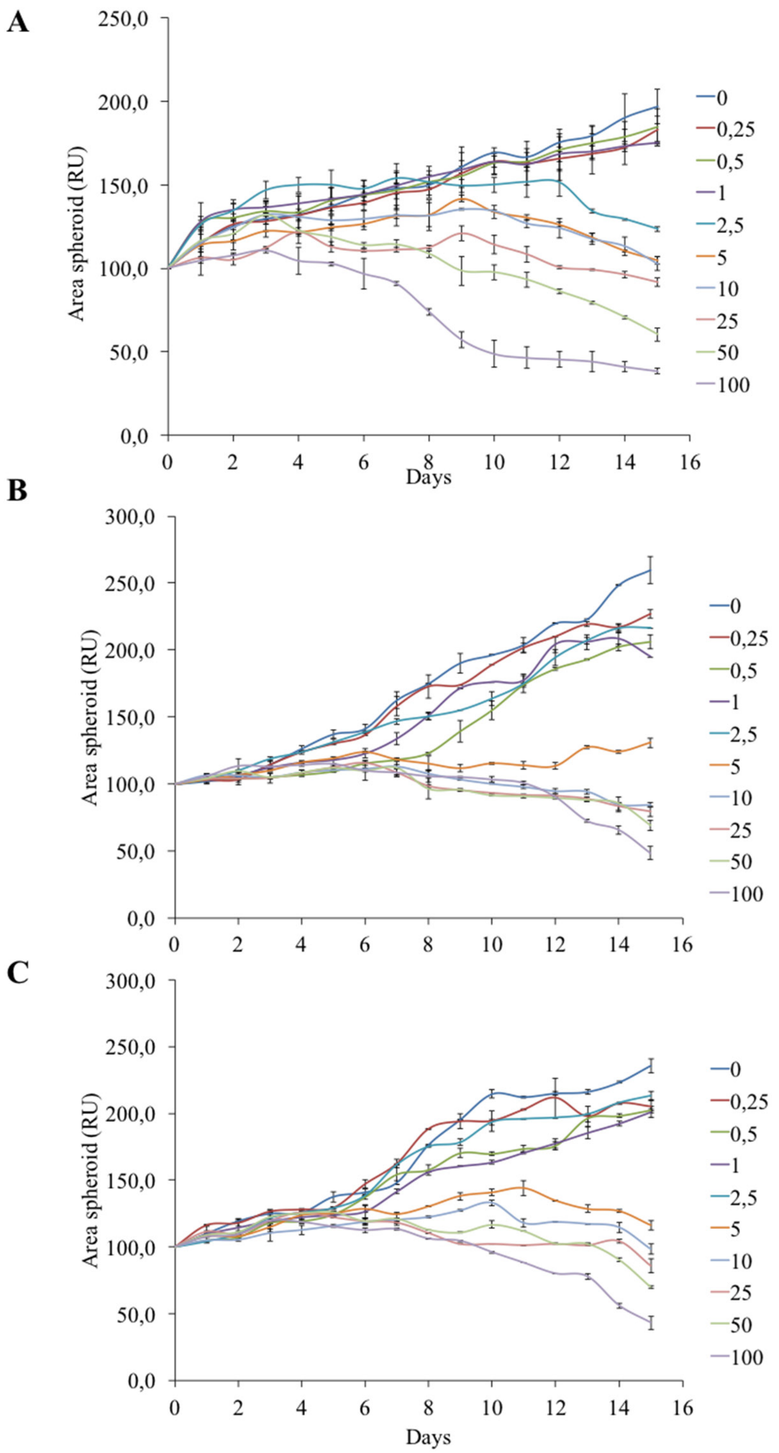

Supplementary Figure 2 : Evolution of spheroid area during 15-day cytotoxicity assays. HT29-D4, Rox1 and Rox2 were seeded in 96-well plates in a medium with 20% of methylcellulose. After a 72-hour incubation allowing the spheroid formation, the cells were treated with increasing concentrations of oxaliplatin (from 0.25 µM to 100 µM). The area were measured every two days for HT29-D4 **(A)**, Rox1 **(B)** and Rox2 **(C)**.

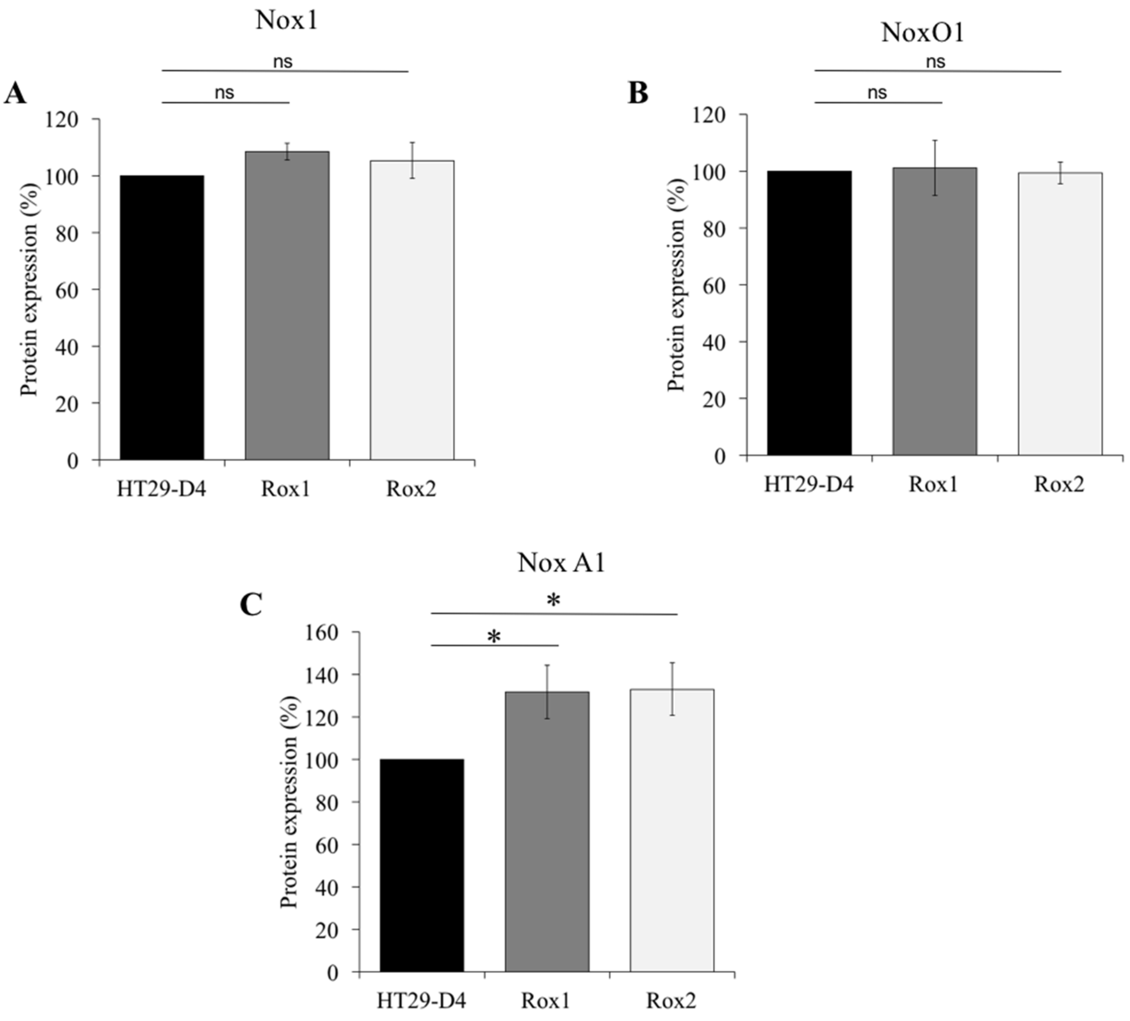

Supplementary Figure 3 : Expression of the components of Nox1 complex. The expressions of Nox1 **(A)**, NoxO1 **(B)** and NoxA1 **(C)** were quantified from the bands obtained by Western blotting. Asteriks indicate a statistical significance with $p<0.05$.

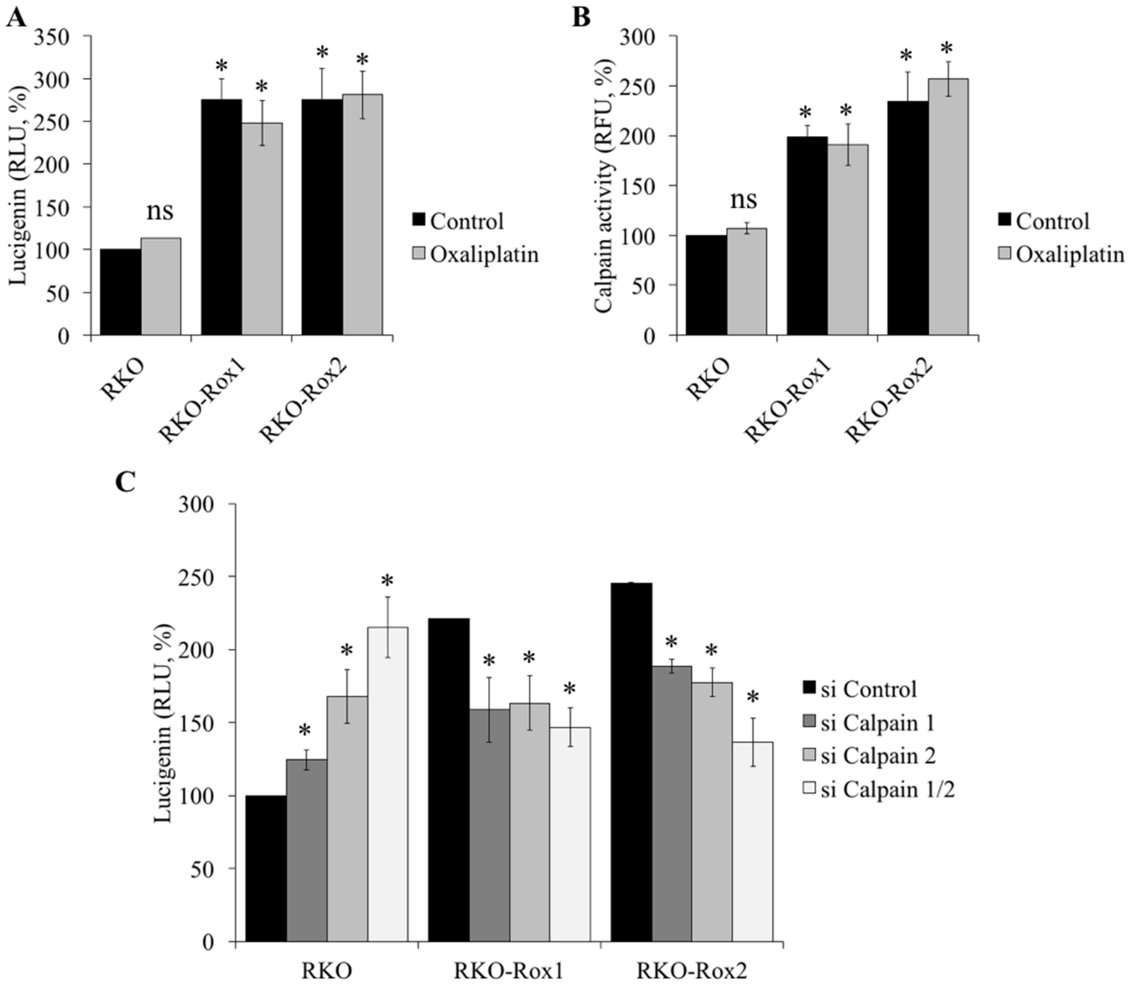

Supplementary Figure 4 : Sensitive and resistant RKO cells behave as sensitive and resistant HT29-D4. RKO, RKO-Rox1 and RKO-Rox2 cells were seeded in white 96-well plates to perform lucigenin assays in the absence (Control) or in the presence of oxaliplatin **(A)**. RKO, RKO-Rox1 and RKO-Rox2 cells were seeded in black 96-well plates to perform calpain activity assays in the absence (Control) or in the presence of oxaliplatin **(B)**. RKO, RKO-Rox1 and RKO-Rox2 cells were transfected with control siRNA (si Control), calpain 1 specific siRNA (si Calpain 1), calpain 2 specific siRNA (si Calpain 2) or both siRNAs (si Calpain 1/2). The cells were seeded in white 96-well plates to perform lucigenin assays **(C)**. Asteriks indicate a statistical significance with $p<0.05$.

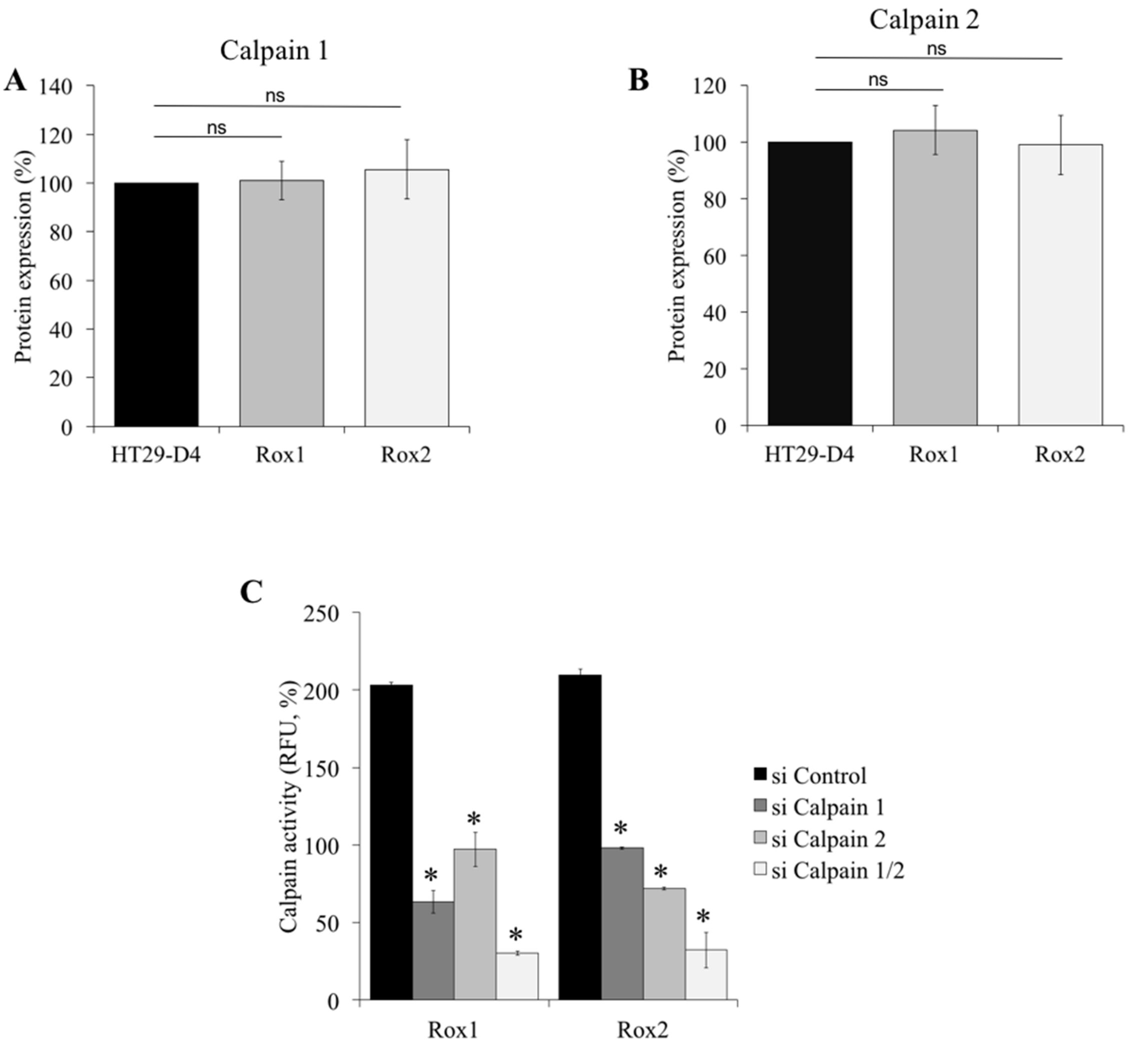

Supplementary Figure 5: Expression and activity of the ubiquitous calpains. The expressions of calpain 1 **(A)** and calpain 2 **(B)** were quantified from the bands obtained by Western blotting. HT29-D4, Rox1 and Rox2 cells were transfected with control siRNA or with siRNAs directed against calpain 1 and/or calpain 2. The cells were seeded on black 96-well plates and calpain activity was measured using t-boc. The activity was expressed as a percentage **(C)**. Asteriks indicate a statistical significance with $p<0.05$.

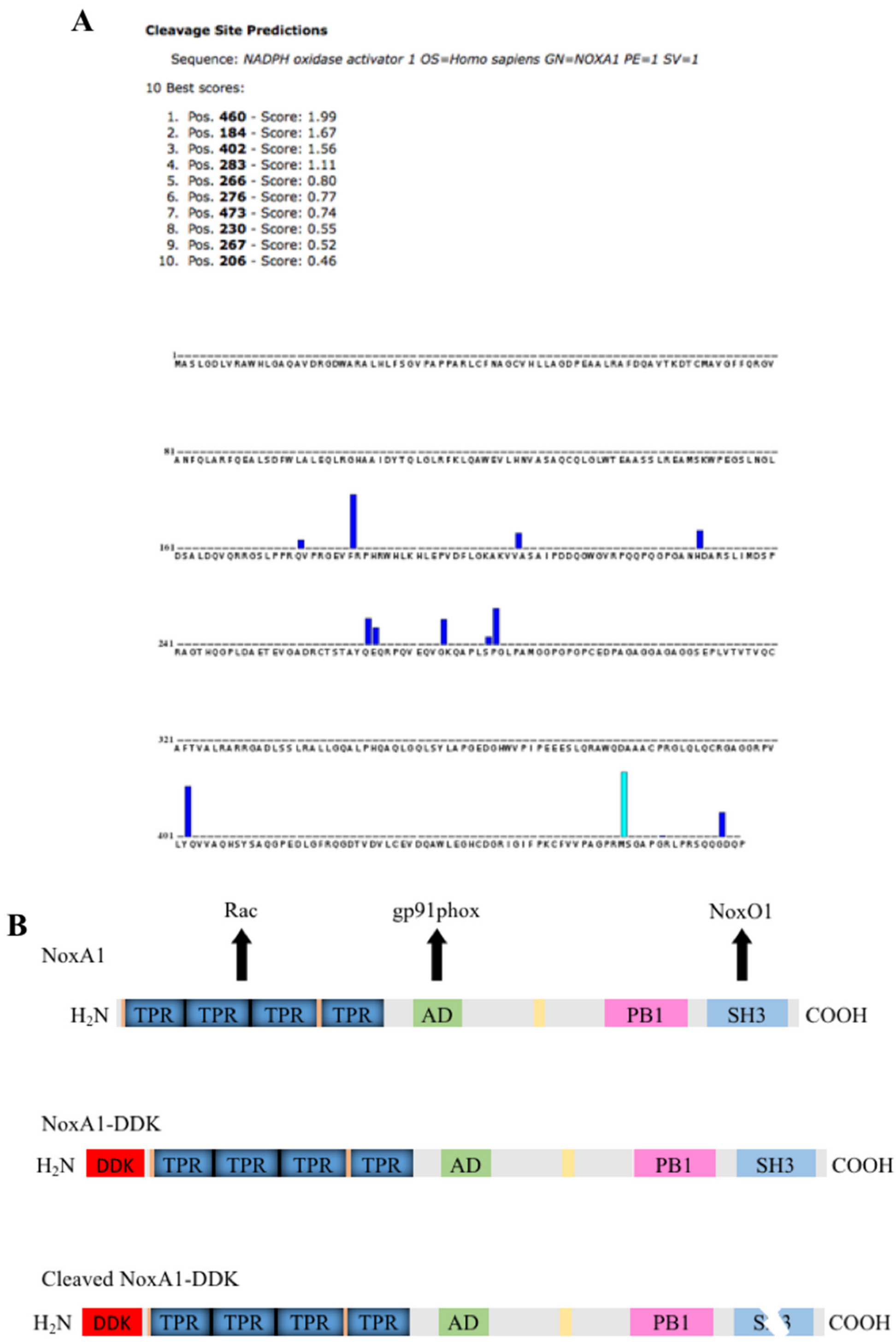

Supplementary Figure 6: NoxA1 is cleaved by calpains in its C-terminal part. Calpain cleavage of NoxA1 was predicted using SVM prediction model (on http://calpain.org/predict.rb?cls=substrate). The cleavage was predicted to occur at the C-terminal end of NoxA1 **(A)**, in the SH3 domain, known to be involved in the interaction with NoxO1 **(B)**.

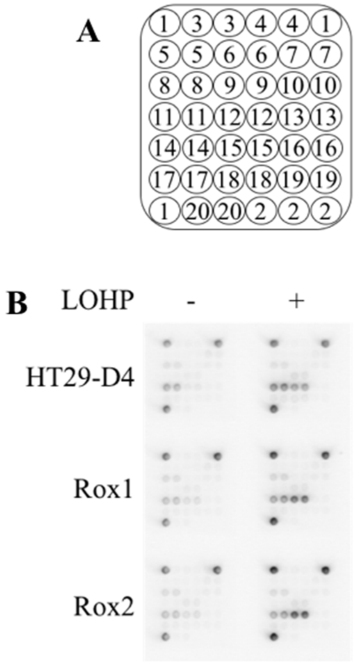

Number	Target	Phosphorylation Site	Modification
1	Positive Control	N/A	N/A
2	Negative Control	N/A	N/A
3	ERK1/2	Ther202/Tyr204	Phosphorylation
4	Stat1	Tyr701	Phosphorylation
5	Stat3	Tyr705	Phosphorylation
6	Akt	Thr308	Phosphorylation
7	Akt	Ser473	Phosphorylation
8	AMPKα	Thr172	Phosphorylation
9	S6 Ribosomal Protein	Ser235/236	Phosphorylation
10	mTOR	Ser2448	Phosphorylation
11	HSP27	Ser78	Phosphorylation
12	Bad	Ser112	Phosphorylation
13	p70 S6 Kinase	Thr389	Phosphorylation
14	PRAS40	Thr246	Phosphorylation
15	p53	Ser15	Phosphorylation
16	p38	Thr180/Thr182	Phosphorylation
17	SAPK/JNK	Thr183/Tyr185	Phosphorylation
18	PARP	Asp214	Cleavage
19	Caspase-3	Asp175	Cleavage
20	GSK-3β	Ser9	Phosphorylation

Supplementary Figure 7: Signaling pathway study using PathScan Array. Target map of PathScan Array allowing the simultaneous detection of 18 important and well-characterized signaling molecules when phosphorylated or cleaved **(A)**. HT29-D4, Rox1 and Rox2 cells were seeded in 6-well plates and were incubated in the absence or in the presence of oxaliplatin for 24 hours. The cells were then lysed and 37.5 µg of cellular proteins were used for the PathScan assay. The raw data were obtained using the G box imaging system **(B)**.

A

n°	Protein kinase name	Specificity score	Normalized kinase statistic
1	p38g MAPK (MAPK12)	2.7	0.42
2	p38b MAPK (MAPK11)	2.7	0.42
3	p38a MAPK (MAPK14)	2.7	0.42
4	CHED	2.7	0.5
5	JNK3 (MAPK10)	2.7	0.43
6	JNK2 (MAPK9)	2.7	0.43
7	JNK1 (MAPK8)	2.7	0.43
8	p38d MAPK (MAPK13)	2.5	0.39
9	ERK5 (MAPK7)	2.4	0.4
10	ERK2 (MAPK1)	1.7	0.36
11	ERK1	1.7	0.36
12	MAK	1.6	0.65
13	CDK3	1.4	0.41
14	CDK1 (CDC2)	1.4	0.41
15	ATR	0.8	0.34

B

n°	Protein kinase name	Specificity score	Normalized kinase statistic
1	p38g MAPK (MAPK12)	1.4	0.6
2	p38d MAPK (MAPK13)	1.4	0.57
3	PRKY	1.1	0.57
4	GSK3A	1	0.53
5	NDR2 (KIAA0965	0.9	0.57
6	PRKX	0.8	0.48
7	p70S6Kb (RPS6KB2)	0.8	0.48
8	DNAPK/PRKDC	0.8	0.56
9	CHK2 (CHEK2)	0.6	0.48

C

n°	Protein kinase name	Specificity score	Normalized kinase statistic
1	p38d MAPK (MAPK13)	2.4	0.34
2	p38b MAPK (MAPK11)	2.0	0.39
3	p38a MAPK (MAPK14)	1.8	0.41
4	JNK3 (MAPK10)	1.8	0.39
5	JNK2 (MAPK9)	1.8	0.39
6	JNK1 (MAPK8)	1.8	0.39
7	p38g MAPK (MAPK13)	1.8	0.36
8	ERK5 (MAPK7)	1.8	0.35
9	ERK1	1.6	0.29
10	CHED	1.5	0.46
11	ERK7 (BI916334)	1.4	0.70
12	ATR	1.4	0.41
13	CDK2	1.2	0.30
14	CDK3	1.0	0.32
15	CDK1 (CDC2)	1.0	0.35

Supplementary Figure 8: Comparison of serine/threonine kinase activities in Rox1 treated with oxaliplatin. Rox1 cells were seeded in 6-well plate and treated with 2 µM of oxaliplatin during 45 minutes, 4 hours and 24 hours. The cells were lysed and 0.5 µg of cellular protein were processed using the Pamgene kinase activity assay. The kinase activities of untreated and treated Rox1 cells were compared. The kinases presenting the strongest modifications after 45 minutes, 4 hours and 24 hours of treatment are presented in (**A**, **B** and **C**), respectively. A positive normalized kinase statistic indicates a kinase activity increased by oxaliplatin treatment.
